# Precious-metal-free rGO/NiMnB nanoarchitectonics with covalent metal support interaction for efficient and durable alkaline water splitting

**DOI:** 10.1186/s40580-025-00516-y

**Published:** 2025-10-28

**Authors:** Shalmali R. Burse, Shamraiz Hussain Talib, Harshitha B. Tyagaraj, S. K. Gagankumar, Swapnil R. Patil, Ebrahim Al Hajri, Jinho Bae, Jungmin Kim, Nilesh R. Chodankar, Yun Suk Huh, Young Kyu Han

**Affiliations:** 1 Department of Energy and Material Engineering, Dongguk University, Seoul, 04620 South Korea; 2 Center for Catalysis and Separations, Khalifa University of Science and Technology, 127788 Abu Dhabi, United Arab Emirates; 3 Mechanical and Nuclear Engineering Department, Khalifa University of Science and Technology, 127788 Abu Dhabi, United Arab Emirates; 4 Department of Biological Sciences and Bioengineering, Nanobio High-Tech Materials Research Center, Inha University, Incheon, 22212 South Korea; 5 Department of Ocean System Engineering, Jeju National University, 102 Jejudaehakro, Jeju, 63243 Republic of Korea

**Keywords:** Transition metal borides, Water electrolysis, Hydrogen and oxygen evolution, Reduced graphene oxide, Statistical analysis

## Abstract

To accelerate the widespread adoption of green hydrogen energy, developing cost-effective, sustainable electrocatalysts that can replace precious metals is essential. This study introduces a bifunctional electrocatalyst for overall water splitting, comprising 2D reduced graphene oxide (rGO) integrated with NiMnB nanosheets into a tailored nanohybrid (rGO/Ni_1.5_Mn_0.5_B). The integration enhances both crystal growth and catalytic activity by promoting rapid nucleation and efficient electron transport. Synthesized via a one-pot hydrothermal process, the rGO/Ni_1.5_Mn_0.5_B electrode exhibits a crystalline 2D nanosheet-like morphology, ensuring high electroactive surface area and strong contact with electrolyte. In 1.0 M KOH, the catalyst achieves an overpotential of 159 mV for hydrogen evolution reaction (HER) and 170 mV for oxygen evolution reaction (OER), outperforming RuO_2_ at 10 mA/cm^2^. DFT calculations reveal that the strong orbital coupling between Ni, Mn, and the rGO matrix enhances metal-support interactions, boosting catalytic performance. The symmetric cell demonstrates overall water splitting cell voltage of 1.49 V at 10 mA/cm^2^ with excellent durability over 20 h under industrial conditions. Additionally, the long-term durability performance was evaluated using time series modelling with a long short-term memory algorithm. With superior electronic, structural, and electrochemical properties, rGO/Ni_1.5_Mn_0.5_B offers a scalable solution for next-generation industrial water splitting and sustainable hydrogen production.

**Graphical abstract:**

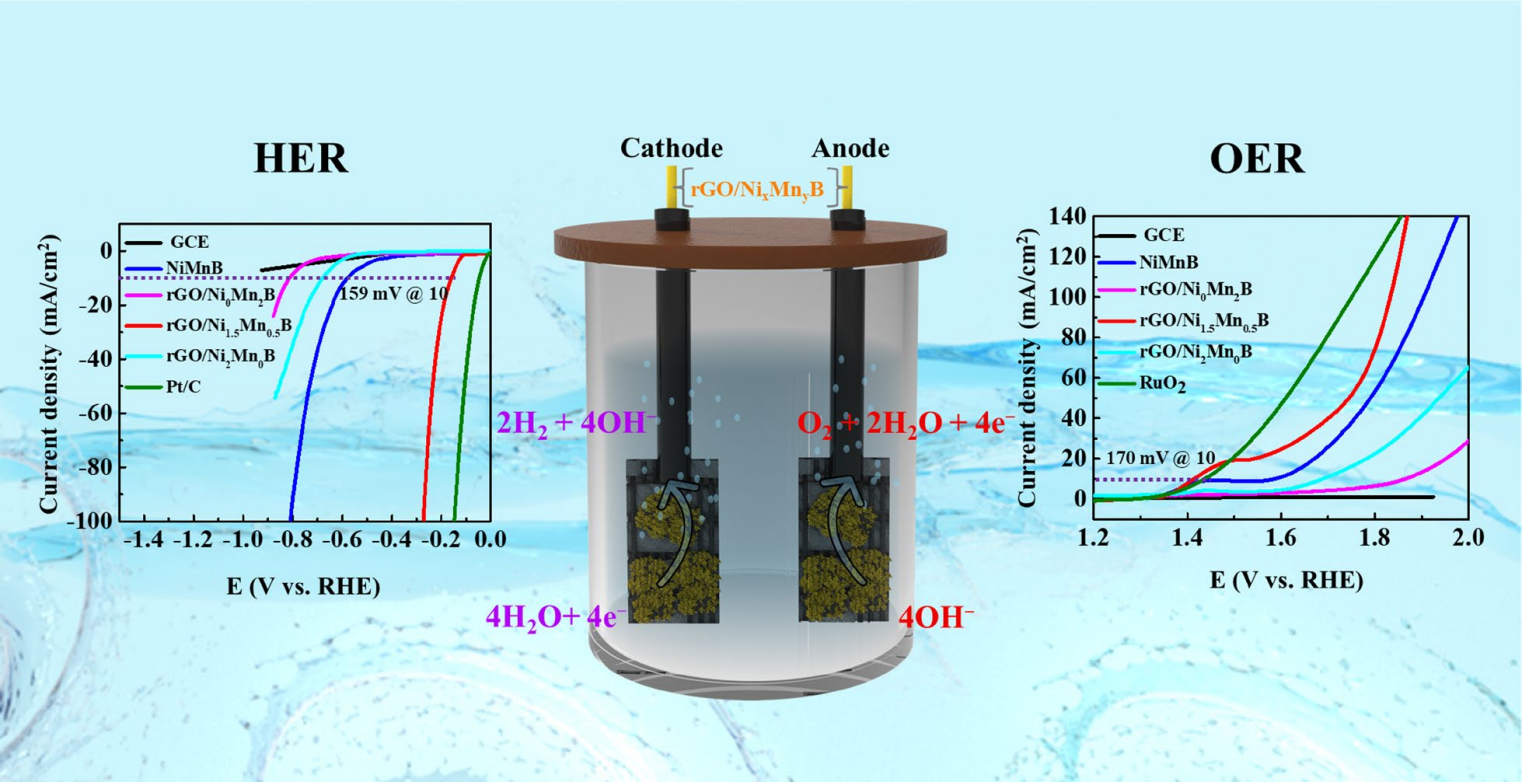
 A bifunctional, precious-metal-free rGO/NiMnB nanohybrid exhibits enhanced alkaline water splitting performance via covalent metal-support interactions. Synergistic orbital coupling at the Fermi level promotes efficient electron transfer, leading to low overpotentials for HER and OER. DFT analysis supports the observed catalytic activity and stability under industrial conditions.

**Supplementary Information:**

The online version contains supplementary material available at 10.1186/s40580-025-00516-y.

## Introduction

Rising environmental and economic concerns about energy have intensified the need for sustainable and efficient technologies. [1–3] Among the various renewable alternatives to fossil fuels, hydrogen (H_2_) stands out as one of the most promising options due to its high energy density and clean combustion. [4–6] These characteristics make it ideal for applications such as hydrogen-powered fuel cells, vehicles, and public transportation systems. [7, 8] Of the various methods for H_2_ production, electrochemical water splitting is considered one of the cleanest and most sustainable approaches. In contrast, over 95% of H_2_ globally is still derived from fossil fuels, with only a small portion produced via water electrolysis. [9, 10] Cost-effective transition metals (TMs) such as Cu, W, Ni, and Fe, along with their derivatives (sulfides, oxides, phosphides, selenides, and borides), have gained significant attention in recent research. [11, 12]These materials are seen as promising and cost-effective alternatives to benchmark noble metals like Pt and RuO_2_ for catalyzing HER and OER. [13–15]

Transition metal borides (TMBs) are considered more significant than other transition metal derivatives due to their unique combination of properties that make them highly efficient and durable in demanding applications. [16–18] In contrast to oxides and hydroxides, which often suffer from poor electrical conductivity, TMBs exhibit metallic-like conductivity, enabling rapid electron transfer. [19] Compared with transition metal phosphides, sulfides, and nitrides that frequently undergo surface oxidation or structural degradation under harsh conditions, TMBs display remarkable chemical robustness and long-term durability. [15] The incorporation of cationic B, characterized by its high electron deficiency, enhances charge transfer between the metal (M) and B, thereby modulating the electronic structure of adjacent metallic sites. [20] This modification improves the electrocatalytic activity. Additionally, their excellent hardness, high melting points, and outstanding chemical stability make them suitable for extreme conditions like high temperatures and corrosive electrolytes. [21] Furthermore, bimetallic TMBs are known for their exceptional conductivity and electron-deficient characteristics, making them promising materials for catalytic applications. [17, 22] Recent advances have revealed that boron-incorporated systems such as Ni_x_B/Mo_0.8_B_3_ nanorods encapsulated by a boron-rich amorphous layer offer abundant active sites and enhanced stability, thereby delivering remarkable bifunctional water splitting performance. [23] These unique merits position TMBs as superior candidates compared to other non-noble metal catalyst systems, making them highly attractive for efficient and durable water-splitting applications. Nickel is an active catalyst for the HER due to its inherent catalytic properties. [24] However, its strong hydrogen adsorption energy (Δ G_H∗_) limits its efficiency. [25, 26] Combining Ni with other metals, such as manganese, can effectively address this issue by modifying the electronic structure and surface properties of Ni to weaken its hydrogen binding and enhance its catalytic activity. [25, 27] Mn reduces the Δ G_H∗_ of Ni and improves the oxophilicity of the material due to its strong affinity for oxygen species. [28, 29] This synergistic combination of Ni and Mn makes Ni-Mn-based materials ideal candidates for developing bifunctional catalysts. Recently, NiMn_2_O_4_ grown on Ni foam showed significant bifunctional activity for HER and OER. [30] Beyond exploring the intrinsic characteristics of Ni-Mn-B, enhancing its catalytic activity has emerged as a focal point in this work. Various methods, like doping of other metals, the introduction of defects, or a combination with conductive substrates like Ni foam, are commonly reported to improve catalytic performance. [31, 32] One of the most promising strategies for improving electrochemical water splitting performance is increasing the catalyst’s electroactive sites. In most cases, enhancing the specific surface area of the catalyst directly contributes to a higher density of electroactive sites, thereby boosting catalytic efficiency. It is well-studied that the rGO has excellent electronic conductivity and a high specific surface area with a 2D sheets-like structure. [33–35] However, the lacking redox activity of the rGO can be overcome by designing the nanohybrid of NiMnB and rGO. For instance, Cu-Ni core–shell nanoparticles anchored on rGO have demonstrated high stability and enhanced catalytic activity as a bifunctional catalyst. [36] Where rGO served as the backbone and provided support to improve the active surface area. [37]

Inspired by the aforementioned concept, rGO/Ni_1.5_Mn_0.5_B nanohybrid is synthesized via a simple one-pot hydrothermal method. This nanohybrid is expected to be a bifunctional catalyst for alkaline water splitting. The optimal Ni:Mn molar ratio was determined to be 1.5:0.5, with the resulting electrode designated as rGO/Ni_1.5_Mn_0.5_B demonstrated significantly enhanced electrocatalytic performance for both the HER and OER, as well as overall water splitting. This improvement can be attributed to its larger specific surface area with abundant exposed active sites, facilitating faster ion transfer and enhancing reaction efficiency. The DFT study reveals that the HER catalytic activity of the nanohybrid arises from the strong covalent coupling between Ni-Mn d-orbitals and B 2p-orbitals at the Fermi level (E_F_), resulting in an optimal |∆G_H*_| value of -0.10 eV, thereby enhancing the HER catalysis. Additionally, the study suggests that the rGO/Ni_1.5_Mn_0.5_B nanohybrid lowers the activation barrier in the potential-limiting step (PDS), improving the OER efficiency. With an enhanced active surface area and strong M‒B synergy, this work makes advanced bifunctional electrocatalytic systems for overall water splitting.

## Experimental section

### Materials and chemicals

Nickel (II) chloride, Manganese (II) chloride tetrahydrate, Ammonium fluoride, and Urea were purchased from Sigma-Aldrich. Boric acid was purchased from Daejung Chemicals and Metals Co. Ltd. Graphene oxide solution was purchased from Grapheneball. All chemicals were of analytical grade and used as received.

### Synthesis of rGO/NiMnB nanohybrid catalyst

**Fig. 1:**
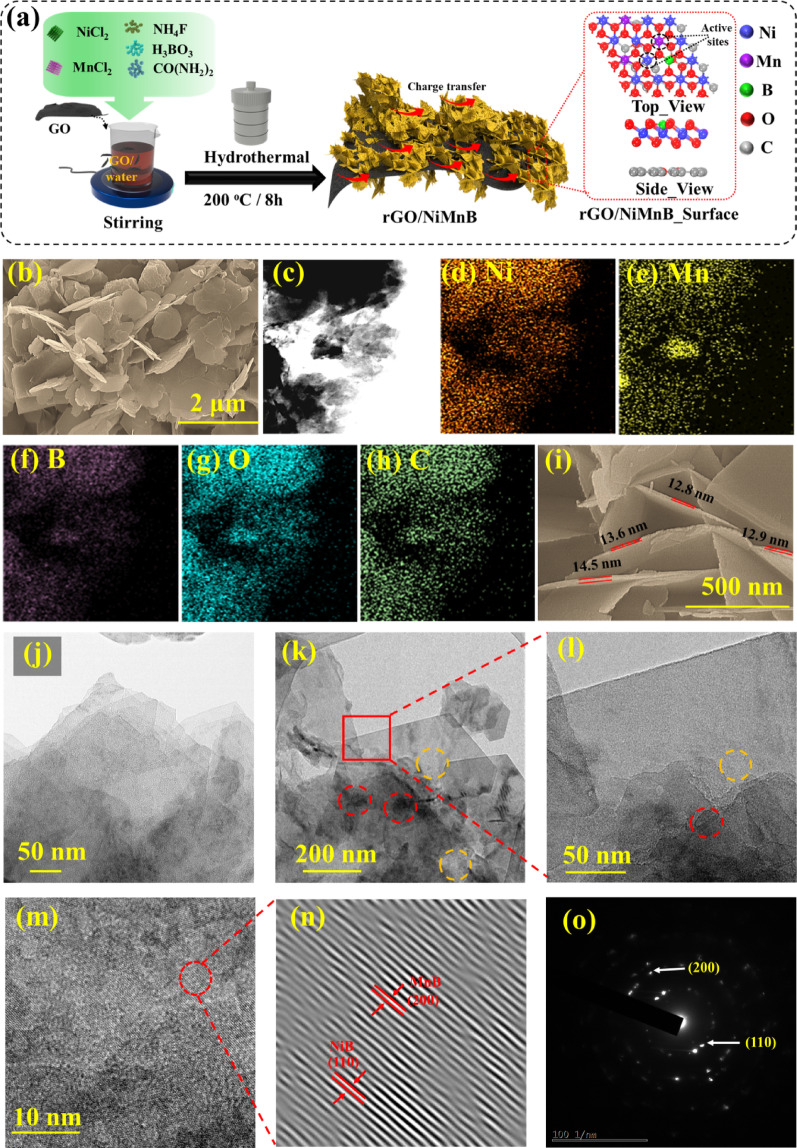
**a** Schematic of the synthesis process of rGO/NiMnB nanohybrids. **b** SEM image of rGO/Ni_1.5_Mn_0.5_B, **c**–**h** Elemental mapping of rGO/Ni_1.5_Mn_0.5_B, **i** HR-SEM image of rGO/Ni_1.5_Mn_0.5_B **j**–**l** TEM images, **m**–**n** HR-TEM images of Ni_1.5_Mn_0.5_, **o** SAED patterns

As illustrated in Fig. 1(a), rGO/Ni_1.5_Mn_0.5_B nanohybrid were fabricated by simple one-step hydrothermal method. Initially, A GO solution was prepared by mixing 7 ml of commercially available GO ink with 70 ml of DI water, followed by stirring at 300 rpm for 2 h. The mixture was then sonicated for 1 h at room temperature, resulting in a well-dispersed suspension. To prepare the rGO/Ni_1.5_Mn_0.5_B nanohybrid catalyst, reaction temperature, reaction time, molar concentration of B and Ni-Mn molar ratio were optimized. The synthesis starts by adding precursors to the previously prepared GO solution. Specifically, 1.5 mM of Ni, 0.5 mM of Mn, 2 mM of B, and specified amounts of NH_4_F and urea were incorporated. Here, urea acted as a mineralizer and etching agent, regulating nucleation and growth of Ni-Mn-B nanostructures, which prevented agglomeration and exposed active sites. Urea served as a slow-release source of OH- ions to control the hydrolysis rate of the metal precursor, ensuring uniform growth, and also helped to reduce GO to rGO during the hydrothermal process. [38] Meanwhile, NH_4_F acted as a morphology-directing agent, where F^−^ ions etched the surface and introduced structural defects, thereby preventing particle agglomeration, exposing active sites, and also improving the interfacial coupling between Ni-Mn-B and rGO. [39] The resulting mixture was sonicated for 30 min to achieve a thorough and uniform dispersion of all components. Following sonication, the prepared solution was transferred into a Teflon-lined autoclave for the hydrothermal reaction. The furnace was set to 200 °C and maintained for 8 h to facilitate the reaction. Upon completion of the hydrothermal process, the product was carefully removed from the autoclave. The resultant material was then subjected to freeze-drying to effectively remove residual moisture and yield a dry powder. Finally, the obtained rGO/Ni_1.5_Mn_0.5_B nanohybrid powder was subsequently used to fabricate electrodes. Similarly, for the comparison NiMnB electrode was fabricated without GO. This detailed preparation process ensured the production of a high-quality rGO/Ni_1.5_Mn_0.5_B nanohybrid powder suitable for various electrochemical applications.

## Results and discussion

The morphological study of rGO/Ni_x_Mn_y_B was analyzed using field emission scanning electron microscopy (FE-SEM) and transmission electron microscopy (TEM). Figure S1 shows the FE-SEM images of rGO. For comparison with optimized electrode, a NiMnB electrode without rGO was fabricated as shown in Figure S2. The SEM image reveals that NiMnB forms an interconnected and aggregated nanosheet-like structure that clusters together. These clusters exhibit a rough and porous morphology, potentially enhancing ion diffusion and electrolyte penetration. Furthermore, the rGO/Ni_x_Mn_y_B nanohybrid was systematically optimized by varying the reaction temperature, reaction time, and molar concentration of B and Ni-Mn ratio, as shown in Figures S3 to S6. Electrochemical analysis details are provided in the next section and in the supporting information. The addition of GO to form rGO/NiMnB nanohybrid introduces several morphological differences and advantages compared to the NiMnB structure alone. The presence of GO not only prevents nanosheet aggregation but also provides a conductive and flexible support that promotes uniform dispersion and growth of active material. Further, the Ni-Mn molar ratio was varied (Ni_0_Mn_2_, Ni_0.5_Mn_1.5_, Ni_1_Mn_1_, Ni_1.5_Mn_0.5_, and Ni_2_Mn_0_) to investigate the effects of Ni-Mn concentration on both morphological and electrochemical performance. While adjusting the Ni-Mn concentration, the total molar concentration was maintained at a constant 2 mM. Changes in precursor concentration ultimately influence the morphology. [40] Figure S6a–d and Fig. 1b represent the morphology of the Ni-Mn concentration ratio variation. The absence of Ni in Figure S6a leads to the formation of aggregated clusters, while the low concentration of Ni (Ni_0.5_Mn_1.5_) in Figure S6b results in uneven growth of nanosheets. The lack of Ni alters the reaction kinetics, and the low Ni concentration affects the nucleation and growth processes, influencing the morphology. [41, 42] In contrast, Ni1Mn1 in Figure S6c shows a transition to more uniform structures, and Ni_1.5_Mn_0.5_ and Ni_2_Mn_0_ in Figs. 1b and S6d exhibit predominant nanosheet structures. The increased Ni content modifies the interactions between metal ions and hydroxide groups, thereby promoting nanosheet growth. [43] This 2D nanosheet structure is expected to provide a high specific surface area, thereby exposing a larger number of active sites for efficient catalysis. [44, 45] The EDX elemental mapping shown in Fig. 1c–h confirms the uniform distribution of all elements throughout the material. Furthermore, the EDS spectrum in Figure S7a displays distinct peaks corresponding to Ni, Mn, B, C, and O, verifying their presence in the sample. The corresponding table provides the atomic and weight percentages of these elements, offering quantitative insight into their composition. Further, the ICP-MS analysis was carried out to identify the exact composition of the optimized sample. Using this analysis, we have identified that the concentrations of Ni, Mn, and B are 75.57, 20.65, and 3.77% respectively consistent with the molar ratios. Furthermore, the high-resolution SEM image in Fig. 1i reveals that the nanosheets have an average thickness of 12–14 nm. The ultrathin nature of the nanosheets minimizes ion and electron diffusion pathways, facilitating faster charge transfer and improving reaction kinetics [46, 47]. Additionally, the interconnected network of nanosheets promotes efficient mass transport, preventing aggregation of active materials and enhancing the overall stability of the catalyst. [48] TEM and high-resolution transmission electron microscopy (HRTEM) analysis were conducted on the optimized rGO/Ni_1.5_Mn_0.5_B nanohybrid for further structural investigation as seen in Fig. 1j-l. The TEM images, shown in Fig. 1k and l, clearly reveal NiMnB nanosheets spread out and attached to rGO surface, forming a unified and efficient nanohybrid structure. The contrast between dark and light areas marked in the figure by a red and yellow dashed circle provides clear evidence of the successful formation of the rGO/Ni_1.5_Mn_0.5_B nanosheet nanohybrid. The dark and light areas could be attributed to the NiMnB and rGO, respectively. The lattice fringe distances of ~ 0.3 nm and ~ 0.2 nm were assignable to the (110) and (200) crystalline planes, confirming the NiB and MnB phases, respectively, as illustrated in Fig. 1m–n. These results are consistent with the XRD analysis, which is discussed in the next paragraph. The selected area electron diffraction (SAED) pattern in Fig. 1o confirms the good crystalline nature of the nanohybrid.

**Fig. 2:**
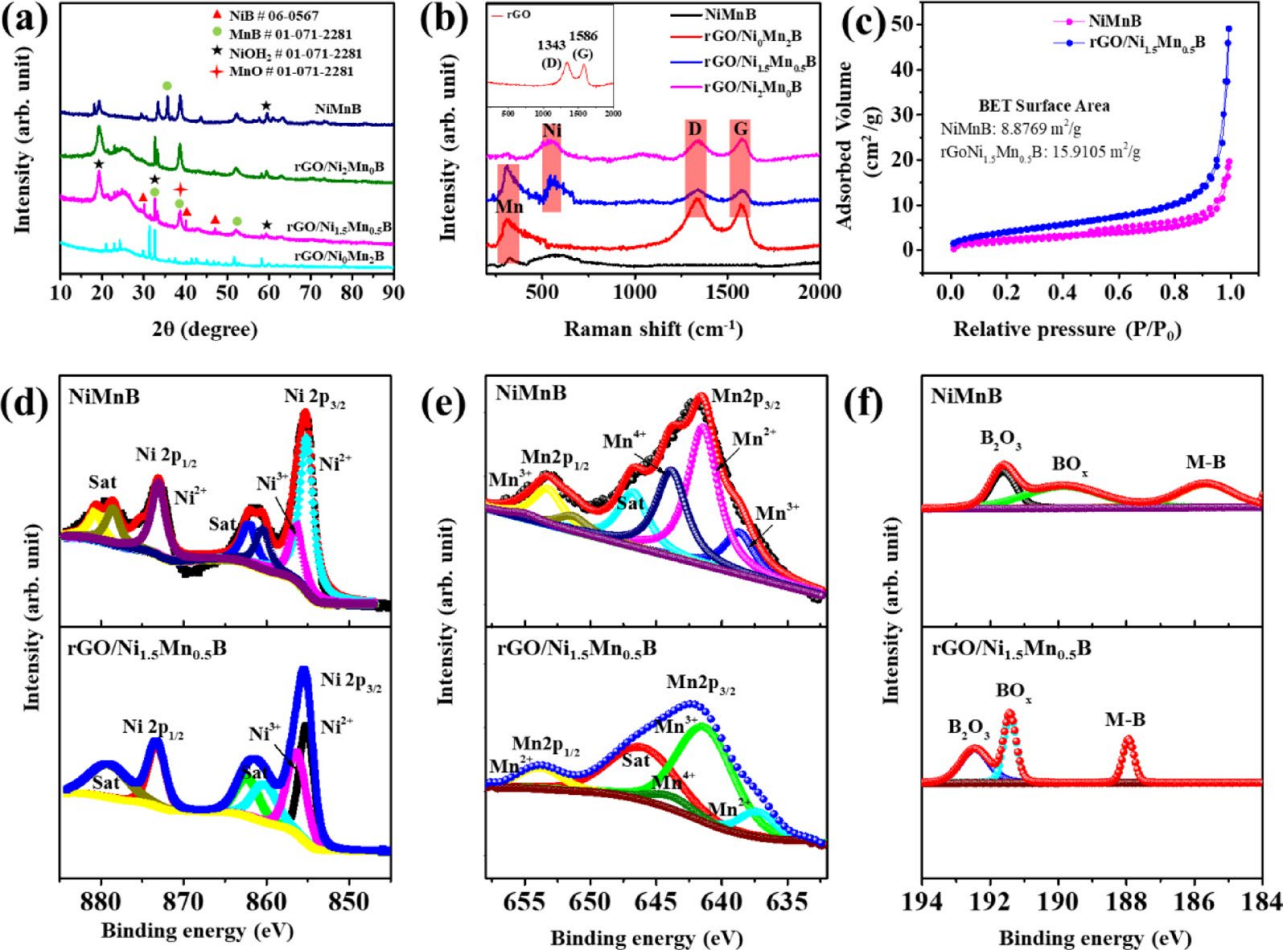
**a** XRD spectra **b** Raman spectra of Ni-Mn concentration variation composites and NiMnB electrode, **c** Nitrogen adsorption/desorption isotherms, **d**–**f** XPS spectra of NiMnB and rGO/Ni_1.5_Mn_0.5_B: Ni 2p, Mn 2p, B 1 s

Further, XRD characterization was employed to identify the phases and crystallinity of as-prepared materials, as shown in Fig. 2a. The XRD pattern of rGO/Ni_1.5_Mn_0.5_B reveals a complex composition with multiple crystalline phases. [49, 50] The NiB phase (reference code 06–0567) is evidenced by peaks at 30.3°, 40.06°, and 47.4°, corresponding to the (110), (021), and (130) planes, respectively. [51] The MnB phase (reference code 01–071-2281) is indicated by peaks at 33.2° (020), 38.5° (200), 52.4° (220) and 35.6° (201). The absence of distinct rGO peaks suggests its low crystallinity compared to Ni-Mn-B, [52] confirming the successful formation of the rGO/NiMnB nanohybrid. Additional phases include nickel hydroxide (reference code 01–073-1520), identified by peaks at 19.2°, 33.2°, and 52.4°, 60.1° corresponding to the (001), (100), (012), and (003) planes. Manganese oxide (reference code 00–044-0992) is indicated by peaks at 38.5° and 59.3°, aligning with the (222) and (511) planes. Notably, peaks at 33.2°, 38.5°, and 52.4° overlap with the MnB phase. The presence of Ni(OH)_2_ and MnO suggests that rGO/Ni_1.5_Mn_0.5_B can function as a bifunctional catalyst, potentially enhancing both the HER and OER. Ni(OH)_2_, known for its high electrochemical activity, particularly facilitates the OER in alkaline media. [25] MnO contributes to both OER and HER, providing additional catalytic sites and structural stability. [53] The synergy between these components, combined with the conductive network of rGO, creates an efficient electrode for electrochemical water splitting. Raman spectroscopy was studied for further analysis. The inset of Fig. 2b displays the Raman spectrum of pristine rGO, exhibiting the characteristic D and G bands at 1343 cm^−1^ and 1586 cm^−1^, respectively. As shown in Fig. 2b, the as-prepared sample shows two prominent peaks at 1342 cm^−1^ and 1584 cm^−1^, matching well with the D and G bands of rGO, confirming the presence of rGO in the nanohybrid. A broad D peak further confirms rGO with a high degree of graphitization. Other prominent peaks were observed at 546 cm^−1^ and 316 cm^−1^, attributed to the Ni–O vibration and Mn–O vibration, [54, 55] respectively. Raman spectra also indicate that B could not be detected due to its low content. The N_2_ adsorption–desorption isotherm and specific surface area of the rGO/Ni_1.5_Mn_0.5_B and NiMnB were analyzed using BET analysis as shown in Fig. 2c. Both exhibit typical H_4_ hysteresis behavior, indicative of a large number of mesopores. In particular, rGO/Ni_1.5_Mn_0.5_B exhibited a prominent hysteresis loop at high relative pressures (0.9 ‒ 1.0 P/P_0_), further confirming the presence of a well-developed mesoporous structure. The surface area of the rGO/Ni_1.5_Mn_0.5_B and NiMnB were determined to be 15.91 m^2^/g and 8.87 m^2^/g, respectively, highlighting the enhanced active sites of rGO/Ni_1.5_Mn_0.5_B nanohybrid. The higher surface area of rGO/Ni_1.5_Mn_0.5_B can be attributed to the integration of rGO, which prevents agglomeration of the active components and introduces additional mesoporosity. The increased surface area ensures a greater number of accessible active sites for catalytic reaction, while the mesoporous architecture facilitates efficient mass transport of reactants and products. [56] This structure minimizes diffusion limitations and enhances the availability of active sites, improving the stability and durability of the catalyst, and making rGO/Ni_1.5_Mn_0.5_B a highly effective material for water-splitting applications.

**Fig. 3:**
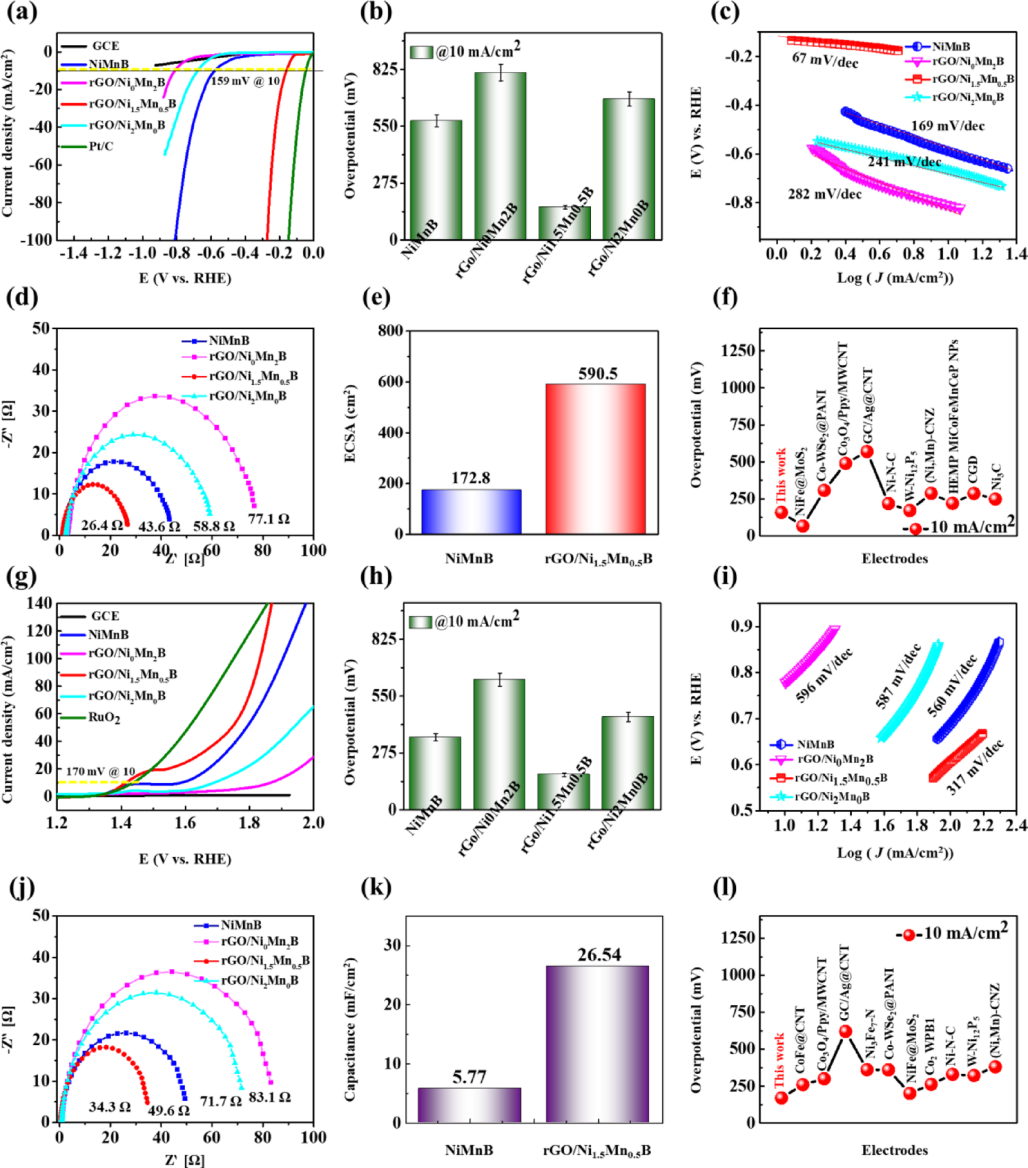
Electrochemical performance study, polarization curve for **a** HER, **g** OER, **b**–**h** Comparison of overpotentials at 10 mA/cm^2^ current density (The error bar represents the range of results from three repeated measurements). **c**–**i** Tafel slopes. **d**–**j** EIS for HER-OER. **e**–**k** ECSA and C_*dl*_ values of NiMnB and rGO/Ni_1.5_Mn_0.5_B electrodes. **f**–**l** Comparison of HER-OER performance of rGO/Ni_1.5_Mn_0.5_B nanohybrid with other reported catalysts in 1 M KOH

X-ray photoelectron spectroscopy (XPS) was performed to analyze the surface elemental composition and chemical state of the rGO/Ni_1.5_Mn_0.5_B nanohybrid and NiMnB electrode in Figs. 3d-f and Figure S8. The survey spectra for the rGO/Ni_1.5_Mn_0.5_B nanohybrid and NiMnB are displayed in Figs. S8a-b respectively show the coexistence of Ni, Mn, O, C, and B for the nanohybrid and Ni, Mn, O, and B for the NiMnB electrode. In the Ni 2p spectrum (Fig. 2d) the two main peaks belonging to the Ni 2p3/2 and Ni 2p1/2 appear at 855.6 and 873.5 eV which were further deconvoluted into six peaks: two peaks at binding energies of 855.2 and 873.3 eV correspond to the Ni^2+^ characteristic spin–orbit doublet [57] while two additional peaks at 856.5 and 874.6 eV correspond to Ni^3+^. In the nanohybrid Ni^3+^ may have a higher intensity compared to NiMnB, which could be caused by stronger interactions with rGO or changes in the local electronic structure induced by B. A positive shift was observed in rGO/Ni_1.5_Mn_0.5_B compared to NiMnB suggesting the increase in the oxidation state of nickel. This shift also highlights the influence of rGO on the electronic structure of the nanohybrid, which directly impacts its catalytic or electrochemical performance. The remaining peaks at 862.5 and 879.3 eV are attributed to the shake-up satellite peaks of nickel. The Mn 2p spectrum was deconvoluted into five distinct peaks in Fig. 2e: two peaks corresponding to Mn^2+^ at binding energies of 637.3 and 653.8 eV, one peak for Mn^3+^ at 641.5, one for Mn^4+^ at 644.2 and a shake-up satellite peak at 646.3 eV. Notably, in the case of the NiMnB electrode, a peak observed at 651.6 eV, attributed to Mn.^2+^ is absent in the rGO/Ni_1.5_Mn_0.5_B nanohybrid. The disappearance of the 651.6 eV peak in the nanohybrid suggests significant changes in the chemical environment and electronic state of manganese when incorporated into the rGO/Ni_1.5_Mn_0.5_B system. Specifically, the absence of this peak indicates that Mn atoms may be participating in an electron transfer process with the rGO. This interaction likely alters the oxidation state distribution of manganese and modifies its coordination environment. [58] The B 1 s spectrum of the rGO/Ni_1.5_Mn_0.5_B nanohybrid in Fig. 2f was deconvoluted into three distinct peaks: borate at 192.4, boron oxide at 191.4, and metal-B at 187.9 eV. Overall, the binding energy of B in the rGO/Ni_1.5_Mn_0.5_B nanohybrid exhibits a positive shift compared to that in NiMnB, indicating electron loss and electron-deficient properties. This behavior effectively inhibits metal oxidation and facilitates the formation of active intermediates. [59] The deconvolution of the O 1 s spectrum in Figure S8c revealed two peaks at 530.7 and 532.3 eV attributed to the C=O and C–OH bonds (absorbed water: H_2_O ads), respectively. [52] The C 1 s spectra from rGO/Ni_1.5_Mn_0.5_B nanohybrid in Figure S8d also deconvoluted into three peaks at a binding energy of 284.4, 285.2, and 288.3 eV attributed to C–C/C=C, C–O, and C=O, respectively. [52]

The intrinsic properties of the optimized rGO/Ni_1.5_Mn_0.5_B and other electrodes, and their electrochemical performance, were evaluated using a standard three-electrode setup in 1 M KOH. In this configuration, the catalyst was coated onto the glassy carbon electrode (GCE) to serve as the working electrode, while Hg/HgO and a platinum electrode were utilized as reference and counter electrodes, respectively. For comparison purposes, the electrochemical performance of commercial Pt/C for HER, RuO₂ for OER, GCE, NiMnB, and rGO/Ni_x_Mn_y_B electrodes was also examined under identical conditions. The GCE, being less conductive, does not contribute significantly to catalytic reactions, enabling a precise evaluation of the intrinsic catalytic properties of the synthesized materials. [60] Fig. 3a presents the HER polarization curves, highlighting the superior performance of the rGO/Ni_1.5_Mn_0.5_B electrode, which achieves an overpotential (η_10_) of 159 mV at 10 mA/cm^2^, indicating excellent catalytic activity for HER in alkaline media. As seen in Fig. 3b the η_10_ value is significantly lower than those of NiMnB (582 mV), rGO/Ni_0_Mn_2_B (814 mV), and rGO/Ni_2_Mn_0_B (683 mV) and is comparable to the Pt/C (51 mV) under the same conditions, which is likely due to the best balance of active sites in the rGO/Ni_1.5_Mn_0.5_B electrode. Also, the rGO/Ni_0_Mn_2_B and rGO/Ni_2_Mn_0_B showed lower performance than the NiMnB due to the following possible reasons: (i) Mn alone (without Ni) is not as active for HER/OER due to its lower catalytic activity and inability to participate effectively in redox reactions critical for these processes. While Mn can enhance stability or conductivity when combined with Ni, catalytic properties are limited in the absence of Ni. (ii) Ni alone might show reduced catalytic performance due to the lack of Mn, which contributes to tuning the electronic structure and stabilizing the intermediate species. [61] (iii) Excessive Ni can lead to agglomeration or structural changes that reduce the number of exposed active sites. These observations confirm that the Ni:Mn ratio plays an important role in improving the catalytic activity. An optimal ratio (Ni_1.5_Mn_0.5_B) can provide the best balance between conductivity and active sites density and intermediate adsorption energy. In this system, Ni offers abundant redox-active sites while Mn modulates the electronic structure, stabilizes reaction intermediates, and prevents excessive Ni agglomeration. [62] However, when Mn is dominant (Ni_0_Mn_2_B), the catalyst suffers from low intrinsic activity and poor electron transfer. On the other side, the Ni-rich system (Ni_2_Mn_0_B) exhibits agglomeration and less adsorption energy. This compositional synergy is also reflected in all other electrochemical performances, as discussed further. The rate kinetic mechanism was studied by measuring the Tafel slope. Figure 3c illustrates the Tafel slope plots used to analyze the HER kinetics of the catalysts. As anticipated, the rGO/Ni_1.5_Mn_0.5_B nanohybrid exhibits a low Tafel slope of 67 mV/dec, suggesting that the catalytic reaction follows the Volmer‒Heyrovsky mechanism: H_2_O + e^−^ → H_ads_ followed by H_ads_ + H_2_O + e- → OH^−^ + H_2_ ↑ where H_ads_ represents the H atom adsorbed on the catalyst surface [63]. Notably, the Tafel slope of rGO/Ni_1.5_Mn_0.5_B is significantly smaller than those of NiMnB (169 mV/dec), rGO/Ni_0_Mn_2_B (282 mV/dec), and rGO/Ni_2_Mn_0_B (241 mV/dec). These lower Tafel slope values indicate the rapid reaction kinetics of the rGO/Ni_1.5_Mn_0.5_B nanohybrid in alkaline media. Moreover, the use of GCE for catalytic performance measurements ensures that the observed activity arises solely from the catalyst rGO/Ni_1.5_Mn_0.5_B itself. [64] In addition, the electrochemical impedance spectroscopy (EIS) measurements were performed to further investigate the charge transfer resistance (R_ct_) of the rGO/Ni_1.5_Mn_0.5_B nanohybrid and other electrodes, as shown in Fig. 3d. The results reveal that while all the electrodes exhibit similar solution resistance (R_s_), their R_ct_ values differ significantly. The rGO/Ni_1.5_Mn_0.5_B nanohybrid demonstrates a notably smaller R_ct_ (26.4 Ω) compared to NiMnB (43.6 Ω), rGO/Ni_0_Mn_2_B (77.1 Ω), and rGO/Ni_2_Mn_0_B (58.8 Ω), highlighting its superior faradaic process and reaction kinetics. This performance is attributed to the rGO sheets, which provide a high surface area and excellent dispersion of NiMnB particles, ensuring enhanced exposure of active sites and efficient electron transport. Additionally, the presence of B improves the adsorption of critical reaction intermediates, further optimizing the energy barrier for the reaction and facilitating faster kinetics. The high surface area and enhanced activity of the rGO/Ni_1.5_Mn_0.5_B nanohybrid are further confirmed by the electrochemical double-layer capacitance (C_dl_), which is directly proportional to the electrochemically active surface area (ECSA). To determine C_dl_, cyclic voltammetry (CV) measurements were performed for NiMnB and rGO/Ni_1.5_Mn_0.5_B during HER processes at various scan rates, as depicted in Figures S9a-b. The linear relationship between current density (ΔJ = Ja-Jc) and scan rate is shown in Figures S10a–b. The rGO/Ni_1.5_Mn_0.5_B nanohybrid exhibits significantly higher C_dl_ and ECSA (23.62 mF/cm^2^, 590.5 cm^2^, respectively) for HER compared to NiMnB (6.91 mF/cm2, 172.8 cm^2^) as shown in Figures S10b and 3e. These findings indicate an enhanced ECSA and a greater number of active sites available in the rGO/Ni_1.5_Mn_0.5_B nanohybrid, thereby contributing to its superior catalytic performance. Table S1 and Fig. 3f present a comparison of the HER overpotential of the synthesized rGO/Ni_1.5_Mn_0.5_B with several recently reported catalysts. The rGO/Ni_1.5_Mn_0.5_B nanohybrid exhibits an overpotential of 159 mV at 10 mA/cm^2^ under alkaline conditions, which is lower than that of several other reported systems, such as Co-WSe@PANI (308 mV), [65] Co_3_O_4_/Ppy/MWCNT (490), [66] GC/Ag@CNT (570), [67] Ni–N-C (218), [68] W-Ni_12_P_5_ (172), [69] (Ni, Mn)-CNZ (288 mV), [70] HEMP NiCoFeMnCrP NPs (220), [71] CGD (287), [72] and Ni_3_C (249). [73] Although some noble-metal-based or specifically engineered materials still show superior activity, the performance of rGO/Ni_1.5_Mn_0.5_B nanohybrid is clearly competitive among non-noble transition metal-based catalysts. The enhanced activity can be attributed to the synergistic interaction between Ni and Mn centers, which modulates the electronic structure and optimizes hydrogen adsorption free energy. Additionally, the conductive rGO framework facilitates efficient charge transfer, while B incorporation further enriches the electronic density near the catalytic sites, thereby accelerating reaction kinetics and improving catalytic efficiency compared to previously reported electrocatalysts.

Following the HER catalytic study, the OER catalytic properties were evaluated at a scan rate of 5 mV/s in 1 M KOH. The polarization curves for OER are displayed in Fig. 3g. Similar to HER, the Ni:Mn compositional effect is also observed for OER. The rGO/Ni_1.5_Mn_0.5_B nanohybrid demonstrates an impressively low overpotential of 170 mV at a current density of 10 mA/cm^2^, outperforming NiMnB, rGO/Ni_0_Mn_2_B, and rGO/Ni_2_Mn_0_B as seen in Fig. 3h. Remarkably, the rGO/Ni_1.5_Mn_0.5_B nanohybrid also surpasses the RuO_2_ benchmark, which exhibits an overpotential of 210 mV at the same current density. The enhanced performance of the rGO/Ni_1.5_Mn_0.5_B nanohybrid can be attributed to the synergistic integration of rGO with NiMnB and the synergistic roles of Ni:Mn. Ni provides highly active redox centers (Ni^2+^/Ni^3+^) that are essential for oxygen evolution, while Mn, with its multiple oxidation states, promotes charge transfer and stabilizes oxygenated intermediates. [74] The balanced Ni: Mn ratio in Ni_1.5_Mn_0.5_B therefore optimizes electron transfer, active site exposure, and intermediate binding, leading to efficient catalytic processes. The nanohybrid exhibits a low Tafel slope of 317 mV/dec and an Rct of 34.3 Ω, as shown in Figs. 3i-j, which highlights its favorable OER kinetics and fast catalytic rate. Further evidence of its superior catalytic properties is provided by the CV measurements (Figures S9e-f), and current density difference plots (Figures S10c-d), which indicate a high electrochemical C_dl_ of 26.54 mF/cm^2^ for the rGO/Ni_1.5_Mn_0.5_B nanohybrid, compared to 5.77 mF/cm^2^ for NiMnB displayed in Fig. 3k. This significantly higher C_dl_ value reflects the nanohybrid’s larger electrochemically active surface area, enabling more active sites to participate in the reaction, thereby enhancing its catalytic efficiency. As shown in Table S2 and Fig. 3l, the rGO/Ni_1.5_Mn_0.5_B nanohybrid requires only 170 mV to reach 10 mA/cm^2^ in alkaline solution, which is the lowest among the compared non-noble metal catalysts. This clearly highlights the superior OER activity of the rGO/Ni_1.5_Mn_0.5_B nanohybrid relative to a wide range of recently developed systems. The designed nanohybrid achieves a balance of efficiency and stability that is competitive with previously reported catalysts. The superior activity arises from Ni-Mn synergy, boron-induced charge enrichment, and a conductive rGO scaffold that promotes efficient charge transfer.

**Fig. 4:**
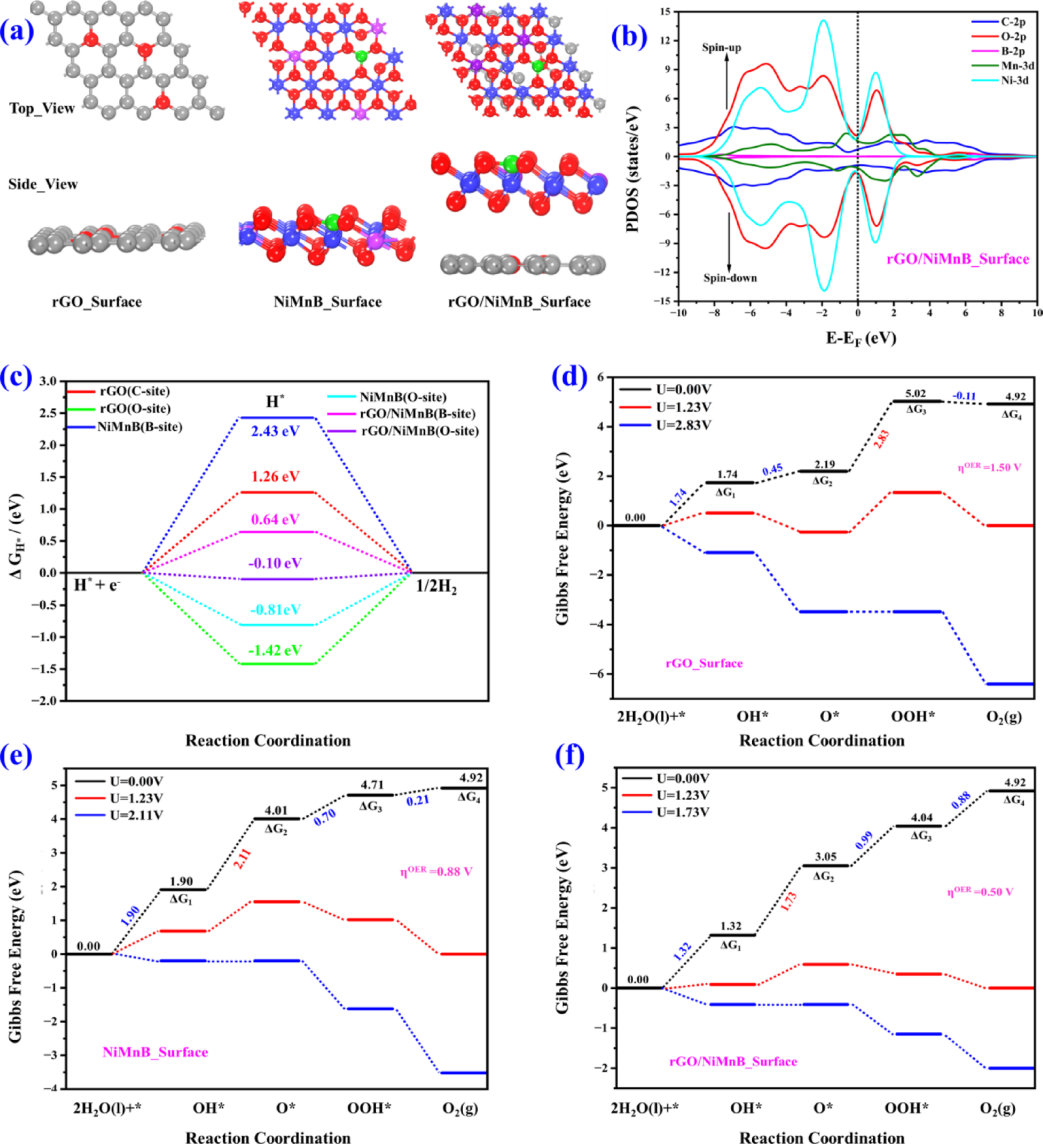
**a** Top and side views of the optimized configuration of rGO, NiMnB, and rGO/NiMnB surfaces. **b** Calculated the partial density of states (PDOS) for the rGO/NiMnB hybrid-structure. **c** The corresponding Gibbs free energy profile (∆G_H*_) for HER at the active site on the rGO, NiMnB, and rGO/NiMnB surfaces. The absolute value of ∆G_H*_ for HER activity is close to zero (∆G_H*_ → 0). **d**–**f** Calculated the free energy profiles of intermediate species on the rGO, NiMnB, and rGO/NiMnB surfaces for OER

We analyzed the HER/OER activities of the synthesized monolayer of rGO and NiMnB surface, and their hybrid structure (rGO/NiMnB) based on the density functional theory (DFT) calculations. We constructed the hybrid-structure (rGO/NiMnB) model based on XPS and TEM results. Figure 4a shows the optimized structures of the proposed models of rGO, NiMnB, and rGO/NiMnB catalysts. The electronic properties of these catalysts were determined using a partial density of states (PDOS) plot. To understand the binding interactions between rGO and NiMnB, the PDOS was projected onto the Mn and Ni 3d orbitals and the C, O, and B 2p orbitals (see Figures S11 and 4b). It is evident from Fig. 4b that the strong coupling between M-d (M = Ni and Mn) orbitals and C, O, and B-2p orbitals at the E_F_ indicates covalent metal-support interactions (CMSI). This symmetric spin-up and spin-down PDOS near the E_F_ indicates that the M-d and C, O and B-2p orbitals have high reactivity, possibly causing the activation of adsorbates during catalysis.

An atomic level first principles-based modeling approach was also used to analyze the active site and HER performance on the rGO, NiMnB, and rGO/NiMnB surfaces. We have calculated the Gibbs-free energies necessary for the adsorption of hydrogen (ΔG_H*_) over rGO and NiMnB surfaces, and rGO/NiMnB hybrid structures (see Fig. 4c). An optimum HER structure must have ΔG_H*_ near to zero (ΔG_H*_ → 0). [75, 76] The result shows that the rGO/NiMnB hybrid structure improved HER performance compared to the monolayer surfaces. Remarkably, the rGO/NiMnB hybrid structure exhibits outstanding catalytic performance in HER, with an optimum |∆G_H*_| value of -0.10 eV, which is consistent with the experimental finding in Fig. 3a.It appears that the rGO/NiMnB hybrid structure is a promising HER catalyst, as its overpotential values are close to zero and like the Pt (111) catalyst (-0.09 V)^1^. Therefore, we conclude that the hybrid structure changes the electronic structure of the surrounding and enhances catalytic HER performance.

The OER activity of stable rGO and NiMnB surfaces and rGO/NiMnB hybrid structures has been investigated, providing insight into their excellent OER performance. Our analysis of OER activity was conducted by computing standard hydrogen electrodes and using self-consistent theoretical overpotential methods. A typical OER process in alkaline conditions comprises four electron steps (see Figures S12-S14) at surface-active sites, with the intermediate products being *OH, O*, *OOH, and O_2_. The intermediates were fascinated by the models of rGO and NiMnB surfaces and rGO/NiMnB hybrid structures. It is evident from the DFT results in Fig. 4d-f that the active sites on these three catalysts are chemically active towards H_2_O, resulting in easy surface oxidation. The Gibbs free energies (ΔG) of OH*, O*, and OOH* species on rGO and NiMnB surfaces and rGO/NiMnB hybrid structures were calculated to identify a rate-determining step (RDS). Based on the four-intensive adsorption/desorption processes and well-defined active sites, equation S2 can be used to determine the appropriate η^OER^ value. The computed overpotential on rGO, NiMnB and rGO/NiMnB surfaces were 1.50, 0.88 and 0.50 V, respectively. As shown in Fig. 4d-f, the RDS of rGO was *O → *OOH, and the RDS of NiMnB and rGO/NiMnB catalyst models was *OH → O*. The calculated overpotential on the rGO/NiMnB catalyst model was 0.50 V intentionally reduced from that of rGO and NiMnB catalysts. Hybrid structures like rGO/NiMnB lead to lower activation barriers in RDS, improving the efficiency of OER. Specifically, this rGO/NiMnB catalyst exhibits excellent catalytic activity, with OER values comparable to those of RuO_2_ (0.42 V)^2^, MoC^2^ (0.45 V)^3^, and IrO_2_ (0.56 V)^2^, further demonstrating that rGO/NiMnB supports the OER process. This indicates that the rGO/NiMnB hybrid structure is worthy of OER support, which is supported by experimental evidence. The comprehensive DFT investigation confirms that the rGo/NiMnB nanohybrid exhibits superior electrocatalytic properties for both HER and OER, demonstrating its potential as an efficient bifunctional catalyst.

**Fig. 5:**
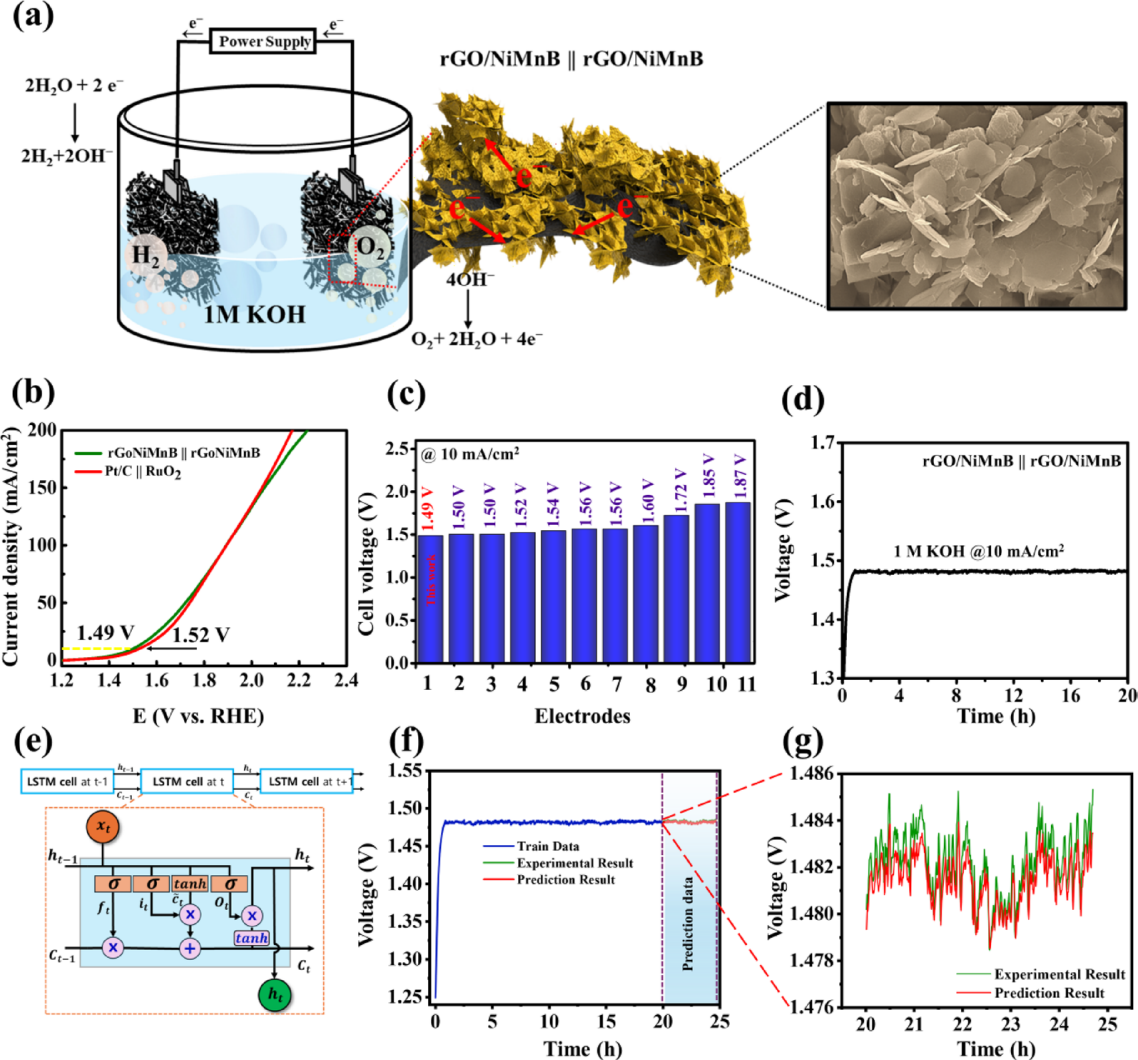
**a** Schematic of overall water splitting. **b** polarization curve of rGO/NiMnB || rGO/NiMnB and Pt/C || RuO_2_, **c** comparison plot of overpotentials between rGO/NiMnB and other reported catalysts for overall water splitting. **d** Long-term stability test in 1 M KOH. **e**Typical structure of LSTM. **f** Experimental, predicted and forecasted stability of rGO/NiMnB||rGO/NiMnB device. **g** Comparison of experimental and LSTM-predicted stability for the rGo/NiMnB||rGo/NiMnB

The results indicate that the rGO/Ni_1.5_Mn_0.5_B nanohybrid demonstrates remarkable electrocatalytic activity for both HER and OER. This suggests its potential as a bifunctional catalyst for overall water splitting, as supported by the computational DFT study. To further validate the suitability of the catalyst for full-cell electrolysis, a symmetric two-electrode configuration was assembled in 1 M KOH solution. The schematic of the developed symmetric cell is presented in Fig. 5a. The polarization curves in Fig. 5b reveal that the rGO/Ni_1.5_Mn_0.5_B nanohybrid requires a cell voltage of only 1.49 V to achieve a current density of 10 mA/cm^2^. This efficiency is outperforming to the benchmark two-electrode system (Pt/C (-) || RuO₂ ( +)), which achieves 1.52 V at 10 mA/cm^2^, and other reported catalysts, as shown in Fig. 5c and Table S3. Furthermore, the rGO/Ni_1.5_Mn_0.5_B nanohybrid-based cell demonstrated excellent stability in Fig. 5d, maintaining its performance over 20 h of testing at a current density of 10 mA/cm^2^. For efficient water-splitting, the stability of the electrode plays an important role. This stability can be attributed to the inclusion of rGO and B, which enhance the active surface area, improve conductivity, and protect against degradation, while B contributes chemical stability and optimizes catalytic performance.

Further, the machine learning-enabled modeling is performed to predict the rGO/NiMnB||rGO/NiMnB device stability. Systematic monitoring of device performance at regular intervals is crucial in energy research, offering deep insights into dynamic behaviors and long-term stability. Advanced time series analysis techniques, such as long short-term memory (LSTM) models, have proven effective in modeling cyclic patterns and predicting device performance. In this study, we utilized an LSTM algorithm to analyze the chrono-potentiometric stability of the rGO/NiMnB||rGO/NiMnB device. The experimental stability results showcase the device’s stability over a 20-h period. To model and predicts the stability of rGO/NiMnB||rGO/NiMnB device, we employed the LSTM algorithm. It is a deep learning technique designed for the prediction of complex data. By leveraging the LSTM model, we can forecast future data trends, providing a more robust framework for assessing and improving the stability and reproducibility of device performance. The LSTM is a specialized deep learning algorithm for time-series classification and sequential data prediction. It is a variant of recurrent neural networks (RNNs) designed to address the problem of long-term dependencies. Unlike conventional RNNs, LSTM incorporates a memory cell that retains and utilizes information over extended time periods, enabling the capture of long-range dependencies. This memory cell updates its state based on current input data and the previous hidden state, selectively preserving or discarding information as needed. Figure 5e shows the structure of LSTM. LSTM is mainly composed of 3 gates. First is the forget gate. In this gate, the sigmoid function is used to determine what information to discard from the cell state. Also, after receiving ${h}_{t-1}$ and ${x}_{t}$ value, send the value between 0 and 1 to ${C}_{t-1}$ using sigmoid function.

1 $${f}_{t} =\sigma ({W}_{f}\cdot \left[{h}_{t-1},{x}_{t}\right] + {b}_{f})$$

where ${h}_{t-1}$ is the last output of the LSTM unit, ${x}_{t}$ is the current input of the current LSTM cell, $\sigma$ is sigmoid function, ${W}_{f}$ is weight and is ${b}_{f}$ bias. From this value from Eq. 1, the next step is to determine which of the new information coming in will be stored in the cell state, following Eq. (2). The sigmoid layer, called the input gate layer, decides the value to update. Then, the tanh function is used to create a vector with values between − 1 and 1 called ${\widetilde{C}}_{t}$ that candidate values following Eq. (3), and is ready to add them to cell state.

2 $${i}_{t} =\sigma ({W}_{i}\cdot \left[{h}_{t-1},{x}_{t}\right] + {b}_{i})$$

3 $$\widetilde{{C}_{t}}=tanh({W}_{C}\cdot \left[{h}_{t-1},{x}_{t}\right] + {b}_{C})$$

where ${W}_{i}$ and ${W}_{C}$ are weight and ${b}_{i}$ and ${b}_{C}$ are biased. After getting value from (2) and (3), the new cell state is updated by multiplying the values of Eqs. 2 and 3, following Eq. (4).

4 $${C}_{t}= {f}_{t}*{C}_{t-1}+{i}_{t}*\widetilde{{C}_{t}}$$

where ${C}_{t-1}$ is the memory cell from the last LSTM unit. The last gate is called the output gate. Here, we need to decide what we extract as output. Therefore, we utilize the sigmoid function to filter values based on the cell state. Further, we apply the tanh function to obtain values between -1 and 1, and multiply them by the filtered value from the sigmoid function, as shown in Eqs. 5 and 6.

5 $${O}_{t} =\sigma ({W}_{O}\cdot \left[{h}_{t-1},{x}_{t}\right] + {b}_{O})$$

6 $${h}_{t}= {O}_{t}*\text{tanh}({C}_{t})$$

where ${W}_{O}$ is weight and ${b}_{O}$ is bias. That result is derived as output and is transferred to the next LSTM unit. Figure 5f represents the experimental, predicted and forecasted stability of the rGO/NiMnB||rGO/NiMnB device. The mean squared error (MSE) between experimental and prediction results is very small (8.45 × 10^–7^). The results clearly indicate that LSTM effectively captures the experimental trend and predicts stability very well. Moreover, the LSTM also forecasts the next 5 h stability of the rGO/NiMnB||rGO/NiMnB device, affirming the model’s ability to track and forecast the operational robustness of the rGO/NiMnB||rGO/NiMnB device (Fig. 5g).

To understand the cause behind the excellent stability of the developed nanohybrid catalyst, we conducted morphological, structural, and compositional analyses after long-term stability test via FESEM, TEM, and XPS, respectively. The corresponding results are presented in Figure S15. The FESEM images in Figures S15a-b show no morphological changes but a slight increase in surface roughness due to prolonged operation. Gas evolution (H_2_ and O_2_ bubbles) can cause localized stress, which slightly alters the surface texture and increases roughness. The post-stability TEM analysis, as shown in Figures S15c-e confirms that the rGO/Ni_1.5_Mn_0.5_B nanosheet nanohybrid retained its morphology after the stability test. The TEM images reveal the continued integration of NiMnB nanosheets with rGO sheets, with no discernible changes in the structural arrangement or nanosheet integrity. The contrast between the dark and light areas in the images, corresponding to NiMnB and rGO, remains consistent, providing evidence that the nanohybrid structure is stable under operational conditions. This morphological stability highlights the robustness of the rGO/Ni_1.5_Mn_0.5_B nanohybrid, making it a durable material for catalytic applications such as OER and HER. Further, the XPS spectra after the stability of rGO/Ni_1.5_Mn_0.5_B are shown in Figures S15f-k. The full-scan XPS spectra of the rGO/Ni_1.5_Mn_0.5_B electrode after the stability test confirmed the presence of all active elements, including Ni, Mn, B, O, and C. High-resolution XPS analysis revealed that the peak positions for these elements remained unchanged, while significant increases in the oxidation states of Ni, Mn, and B were observed. The high-valent state of metallic Ni was found to promote the formation of Ni-OOH, a key intermediate species known to serve as an active site for the OER. [77, 78] The Mn 2p spectra indicated a shift towards higher oxidation states, reflecting the transformation of Mn into Mn^3+^ and Mn^4+^ species. [79] These higher oxidation states of Mn are critical for enhancing the catalytic activity of the electrode. [78] These higher-valent Mn species are known to play a dual role in catalysis: stabilizing intermediate species during OER and facilitating electron transfer during HER. [78] This dual functionality makes Mn an integral contributor to the overall performance of the electrode. The B 1 s spectrum showed a marked increase in the intensity of the B_2_O_3_ peak, which is attributed to surface oxidation during the stability test. The oxidized boron species may influence HER by modulating the local electronic structure, thus optimizing hydrogen adsorption energies on the electrode surface. The O 1 s spectra demonstrated the formation of additional oxygen species, likely from surface hydroxyl groups (-OH) and metal oxides, essential for improved OER performance. This suggests the generation of active oxygen-containing compounds during the stability test. Additionally, they may enhance HER by providing favorable sites for hydrogen adsorption, aiding the Volmer step in the HER mechanism. The C 1 s spectra highlighted the presence of sp^2^-bonded carbon (C=C) and carbonyl group (e.g., C=O), originating from the reduced graphene oxide (rGO) matrix. The rGO matrix not only provides a highly conductive framework for efficient charge transfer but also offers partial hydrophilicity due to oxygen-containing functional groups. This hydrophilicity promotes the adsorption of hydrogen and oxygen intermediates, enhancing HER and OER performance. XPS analysis post-stability demonstrates that the synergistic effects of Ni, Mn, and B, coupled with the structural and electronic contributions of the rGO matrix, create an optimized electrode surface. The observed increases in oxidation states and surface modifications collectively enhance the catalytic efficiency and stability of the rGO/Ni_1.5_Mn_0.5_B electrode for both OER and HER, making it a promising bifunctional catalyst for water-splitting applications.

## Conclusion

In summary, rGO/NiMnB nanosheet nanohybrid bifunctional electrocatalysts were successfully synthesized via an in situ one-pot hydrothermal process. Among all synthesized electrodes, the rGO/Ni_1.5_Mn_0.5_B nanohybrid exhibited outstanding HER activity, achieving 159 mV at 10 mA/cm^2^, while in OER, it outperformed the RuO_2_ benchmark electrode with an overpotential of 170 mV at 10 mA/cm^2^. The symmetric cell setup for overall water splitting demonstrated a low cell voltage of 1.49 V at 10 mA/cm^2^, with excellent stability, maintaining continuous performance over 20 h in chronopotentiometry. Additionally, long-term stability was confirmed through statistical modeling and LSTM techniques. Theoretical calculations further revealed that the rGO/NiMnB hybrid structure exhibits strong covalent metal-support interactions, optimizing the electronic structure and enhancing catalytic activity. The optimized Gibbs free energy (∆G_H*_ = − 0.10 eV) indicates superior HER performance, while the reduced activation barriers in the PDS significantly improve OER efficiency. These findings highlight the rGO/NiMnB nanosheet nanohybrid as a highly efficient, stable bifunctional electrocatalyst, making it a promising candidate for sustainable energy applications. While challenges such as large-scale synthesis and long-term operation under industrial conditions remain, the excellent performance demonstrated here provides a solid foundation for future development. With further optimization and integration into practical devices, rGo/NiMnB-based systems hold significant potential for advancing scalable, sustainable hydrogen production technologies.

## Supplementary Information

Below is the link to the electronic supplementary material.

Supplementary Material 1

## Data Availability

All data generated or analyzed during this study are included in this article.

## Abbreviations

rGO: Reduced graphene oxide
HER: Hydrogen evolution reaction
OER: Oxygen evolution reaction
TMs: Transition metals
TMBs: Transition metal borides
M: Metal
Δ G_H__∗_: Adsorption energy
E_F_: Fermi level
PDS: Potential-limiting step
FE-SEM: Feld emission scanning electron microscopy
TEM: Transmission electron microscopy
HRTEM: High-resolution transmission electron microscopy
SAED: Selected area electron diffraction
XRD: X-ray diffraction
XPS: X-ray photoelectron spectroscopy
GCE: Glassy carbon electrode
EIS: Electrochemical impedance spectroscopy
C_dl_: Double-layer capacitance
CV: Cyclic voltammetry
DFT: Density functional theory
PDOS: Partial density of states
CMSI: Covalent metal-support interactions
RDS: Rate-determining step
LSTM: Long short-term memory
RNNs: Recurrent neural networks
MSE: Mean squared error
