# Supplementary material for: Precious-metal-free rGO/NiMnB nanoarchitectonics with covalent metal support interaction for efficient and durable alkaline water splitting
Source: Nano Converg. 2025 Oct 28;12:50. doi: 10.1186/s40580-025-00516-y (PMC12569245; doi:10.1186/s40580-025-00516-y)
Supplement: Supplementary file 1 — Supplementary Material 1 [file 40580_2025_516_MOESM1_ESM.docx]

**Supporting Information**

**Precious-Metal-Free rGO/NiMnB Nanoarchitectonics with Covalent Metal Support Interaction for Efficient and Durable Alkaline Water Splitting**

*Shalmali R Burse^a^, Shamraiz Hussain Talib^b^, Harshitha B Tyagaraj^a^, Gagankumar S K^a^, Swapnil R Patil^d^, Ebrahim Al Hajri^c^, Jinho Bae^e^, Jungmin Kim^e^, Nilesh R. Chodankar^c^*, Yun Suk Huh^d^*, Young Kyu Han^a^**

*^a^Department of Energy and Material Engineering, Dongguk University, Seoul, 04620, South Korea*

*^b^Center for Catalysis and Separations, Khalifa University of Science and Technology, Abu Dhabi, 127788, United Arab Emirates*

*^c^Mechanical and Nuclear Engineering Department, Khalifa University of Science and Technology, Abu Dhabi, 127788, United Arab Emirates.*

*^d^Department of Biological Sciences and Bioengineering Nanobio High-Tech Materials Research Center, Inha University, Incheon 22212 South Korea.*

*^e^Department of Ocean System Engineering, Jeju National University, 102 Jejudaehakro, Jeju 63243, Republic of Korea*

*Corresponding author: [nilesh.chodankar@ku.ac.ae](mailto:nilesh.chodankar@ku.ac.ae), [yunsuk.huh@inha.ac.kr](mailto:yunsuk.huh@inha.ac.kr), [ykenergy@dongguk.edu](mailto:ykenergy@dongguk.edu)

**Electrode fabrication**

A 3 mm glassy carbon electrode (GCE) was used for electrode fabrication. To prepare the nanohybrid ink, 5-10 mg of rGO/Ni_1.5_Mn_0.5_B nanohybrid powder was dispersed in a mixture containing 390 μL isopropanol, 600 μL water, and 10 μL Nafion (as a binder). The suspension was sonicated for 10 -15 minutes. Subsequently, 5 μL of this nanohybrid ink was drop-cast onto the GCE surface using a micropipette. After drying, the electrochemical performance of the electrode was evaluated in a 1 M KOH solution. Similarly, various electrodes prepared with varying Ni-Mn concentration ratios (0:2, 0.5:1.5, 1:1, and 2:0)

**Physical, structural and electrochemical characterization**

To analyze the physical morphology of the rGO/Ni_x_Mn_y_B samples, a field emission scanning electron microscope (HR-SEM, HITACHI S-4800, Japan) was employed. To investigate the internal structure, a high-resolution transmission electron microscope (HR-TEM, JEM-2100F, JEOL, Japan) equipped with an energy-dispersive X-ray spectroscope (EDS) was used to analyze the elemental composition. Inductively coupled plasma optical emission spectroscopy (ICP-OES, 7300DV PerkinElmer, USA) was carried out to detect trace elements. X-ray diffraction (XRD, X'Pert-PRO MRD, Philips, The Netherlands) using Cu Kα irradiation and X-ray photoelectron microscopy (XPS, Thermo Fisher Scientific, K-alpha, USA) were used to examine the phase structure and surface chemistry of the rGO/Ni_x_Mn_x_B samples. Raman spectroscopy was performed using a laser Raman spectrometer (Raman, FEX, NOST, Republic of Korea) with a 532 nm excitation source. The surface area was determined by Brunauer–Emmett–Teller (BET) measurement, and the density functional theory (DFT) calculations were conducted using the Vienna Ab initio Simulation Package (VASP)

**Computational Details**

Calculations based on first-principles DFT, including molecular dynamics simulations were executed utilizing the VASP [1] in conjunction with the Projector Augmented Wave (PAW) methodology [2]. The exchange-correlation functional was managed within the parameters of the Generalized Gradient Approximation (GGA), adopting the Perdew-Burke-Ernzerhof (PBE) functional [3]. The long-range van der Waals interactions are accounted for through the DFT-D3 approach [4]. For details of model building, rGO surface, NiMnB surface and rGO/NiMnB hybrid structure. We implemented a plane wave basis set with an energy cutoff set at 450 eV, and the geometric relaxation was carried through until the forces acting on each atom were less than 0.03 eV/Å. The sampling of the Brillouin zone was conducted using a 2 × 2 × 1 k-point grid. To assure rigorous consistency, calculations were performed until the energy convergence threshold was less than 10^-5^ eV. To effectively isolate periodic structures and preclude their interaction, a vacuum buffer of 15 Å was inserted along the z-axis.

 The free energy of the intermediates is calculated：

ΔG = ΔE_DFT_ + ΔE_ZPE_ – TΔS (S1)

where ΔE_DFT_, ΔE_ZPE_ and ΔS are the changes of the reaction energy obtained from DFT calculations, zero-point energy, and the changes of entropy from the initial state to the final state, respectively. T is temperature and the T of 298.15 K was used in all computations.

Gibbs free energy (∆G) for four elementary steps for OER processes is defined as follows: ∆G_a_ = ∆G­_OH*_, ∆G_b­_ = ∆G­_OH*_ - ∆G­_O*_, ∆G_c_ =_­_ ∆G­_O*_ - ∆G­_OOH*_, ∆G_4­_ = ∆G­_OOH*_ - 4.92.

Using the overpotential (η), OER catalytic activity can be further justified if all four steps have different Gibbs free energy values. The overpotential can be calculated using the following equation.

η ^OER^ = max {∆G_a_, ∆G_b­_, ∆G_c­_, ∆G_4d_}/e -1.23 (S2)

**Electrochemical Study**

For electrochemical performance, CHI 7089E electrochemical workstation was used with a three electrode system. In this system, the Hg/HgO electrode was used as a reference electrode, the Platinum electrode was used as the counter electrode, and the synthesised rGO/NixMnyB material-coated GCE was used as a working electrode. For the electrochemical performance of all the fabricated electrodes, linear sweep voltammetry results (LSV) were taken without IR correction. The benchmark electrodes of Pt/C and RuO_2_ were used for the comparison. All the measurements were carried out in the 1 M KOH as alkaline. All 3-E potentials were converted to a reversible hydrogen electrode (RHE) based on the following relation: E [V vs RHE] = E(Hg/HgO) + 0.1971+ 0.059 × pH (14). Polarization curves were generally obtained by the LSV with a scan rate of 5 mV/sec in a potential range between 0.2 V and -0.6 V versus RHE for the hydrogen evolution reaction (HER) and 1.1 to 2.2 V for the oxygen evolution reaction (OER) in 1 M KOH. Further, the Tafel slope is obtained from the linear range of LSV plots based on the η = a + b log(j), where the η: overpotential, a: Tafel constant, b: Tafel slope, and j: current density. The electrochemical impedance spectroscopy (EIS) was conducted at varied voltages corresponding to the fixed current density of 10 mA/cm^2^ between 100 kHz and 0.1 Hz with an amplitude of 5 mV. Considering the R_ct_ variation at different applied voltages, the 10 mA/cm^2^ corresponding voltages were used for the EIS measurement around the catalytic turnover region. CV curves were utilized to calculate double-layer capacitance (*C_dl_* ) values of different electrodes. CV curves were performed at a range of scan rate 20 to 140 mVs-^1^ in the non-faradic region. More details related to the C*_dl_* and anodic and cathodic current calculated by the equation *∆j* = (*Ja* - *Jc*)/2 can be found in Figs. S9-S10. The electrochemically active surface area (ECSA) was obtained according to ECSA = $\frac{C_{dl}}{C_{s}}$, where Cs is surface capacitance = 40 µF cm^-2^.  [5]

**
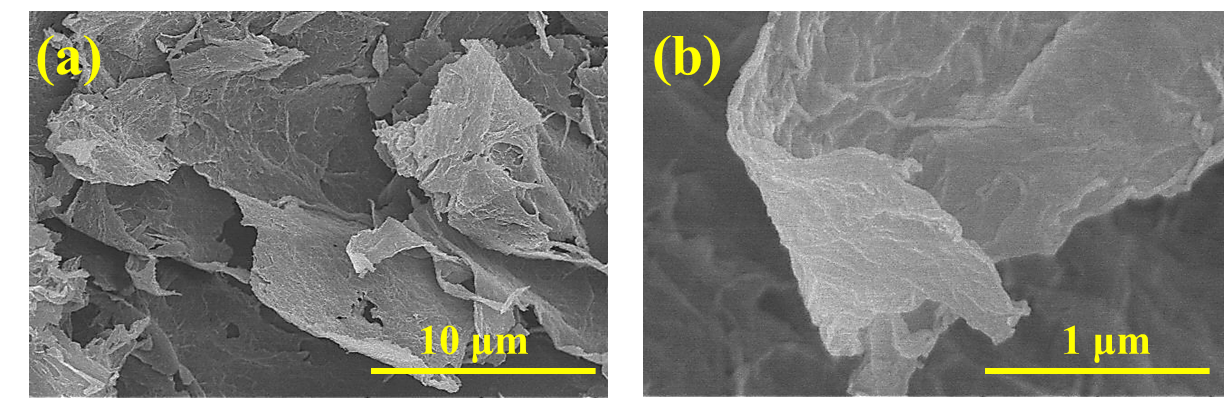
Fig. S1.** (a)-(b) FE-SEM images of rGO.

**
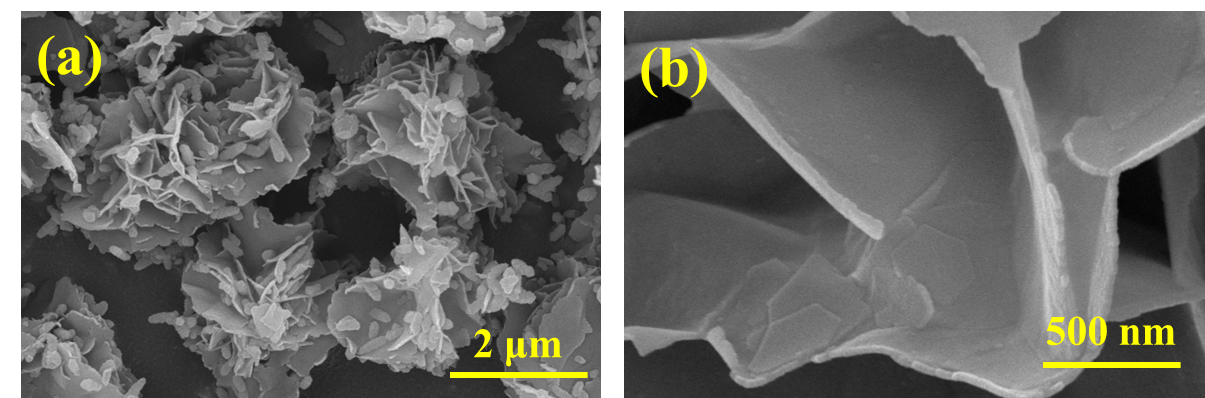
Fig. S2.** (a)-(b) FE-SEM images of NiMnB electrode.

**
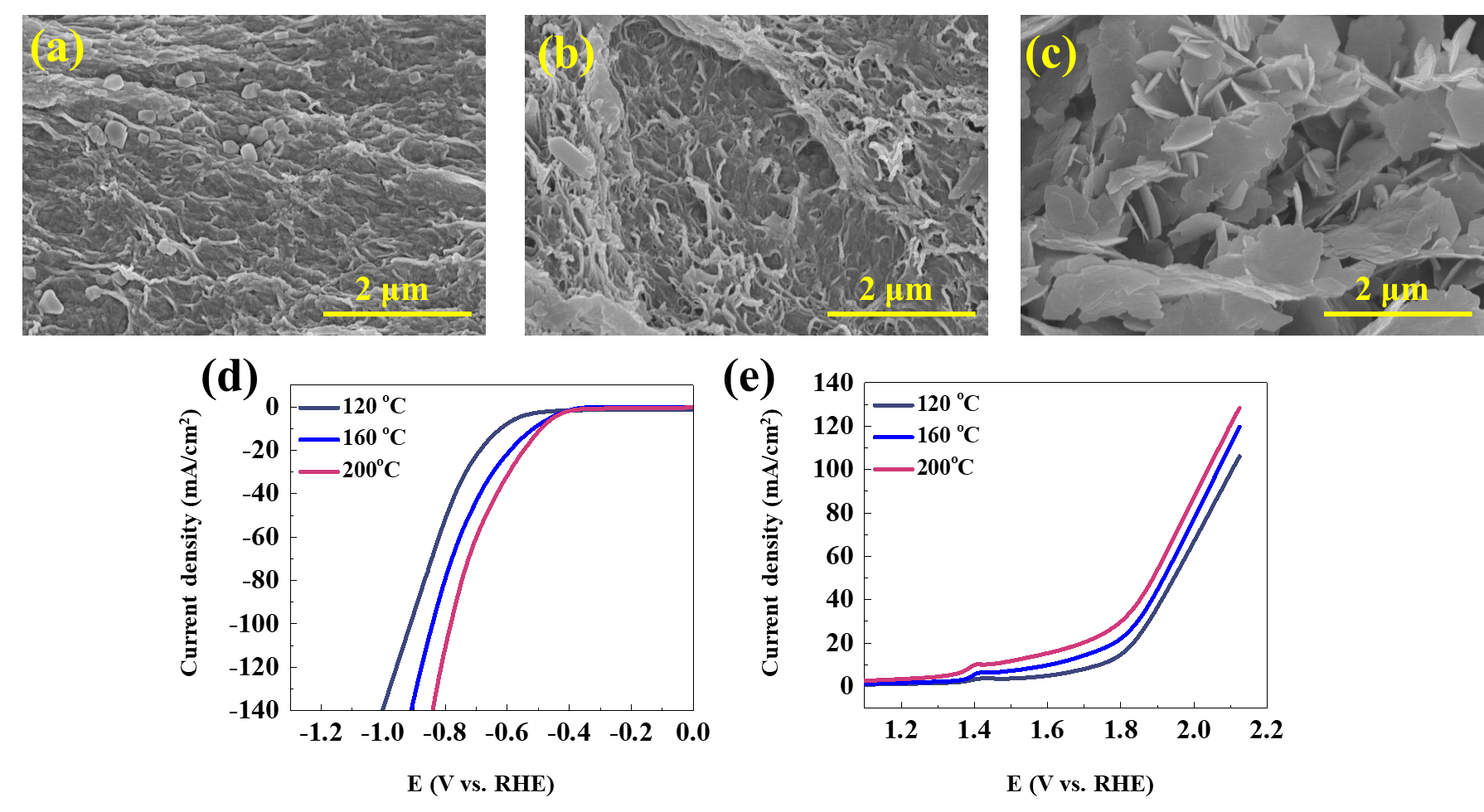
Fig. S3.** (a)-(c) FE-SEM images of reaction temperature variation. (d)-(e) corresponding electrochemical performance for HER, OER.

**
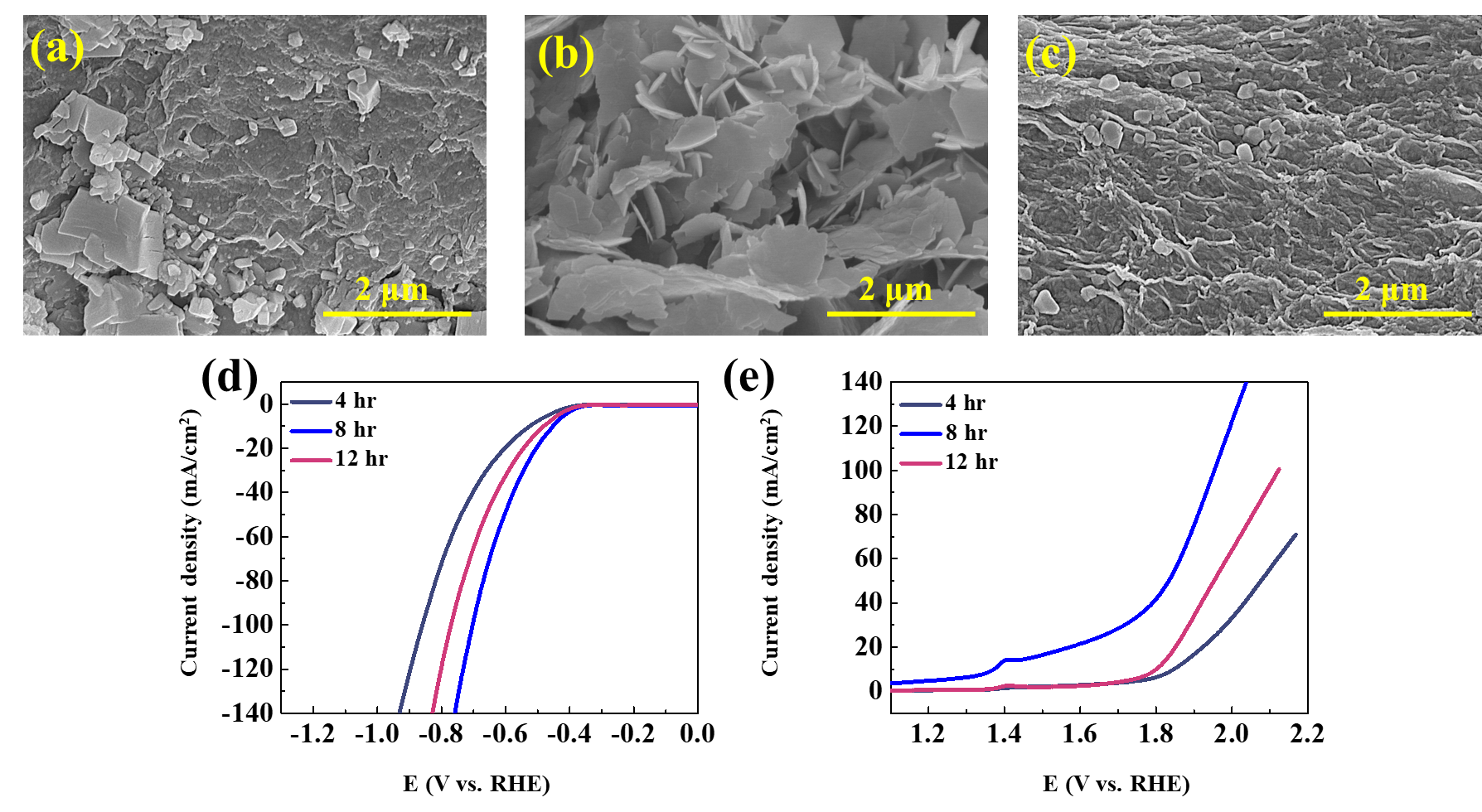
Fig. S4.** (a)-(c) FE-SEM images of reaction time variation. (d)-(e) corresponding electrochemical performance for HER, OER.

**
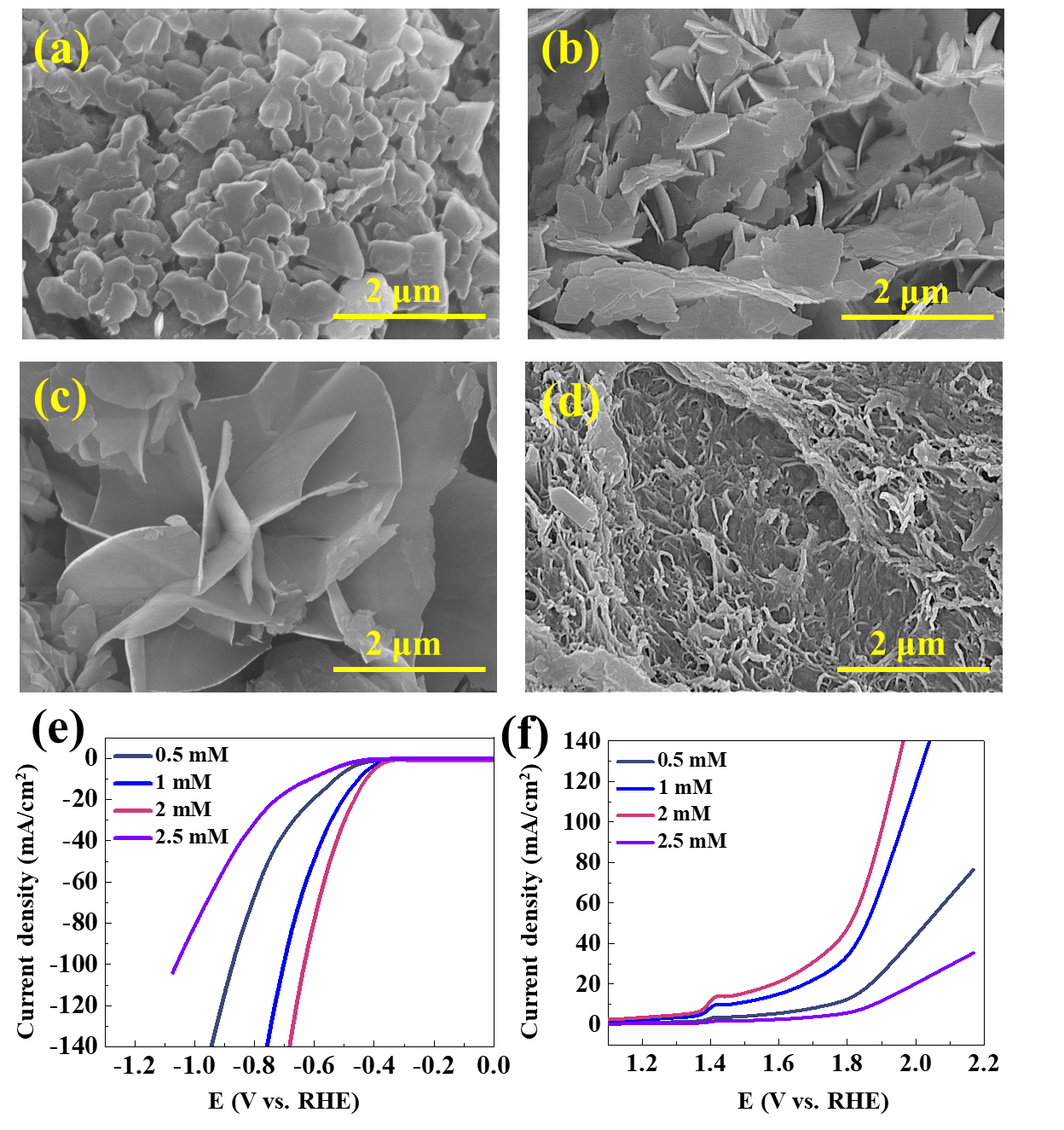
**

**Fig. S5.** (a)-(d) FE-SEM images of boron concentration variation. (e)-(f) corresponding electrochemical performance for HER, OER.

**
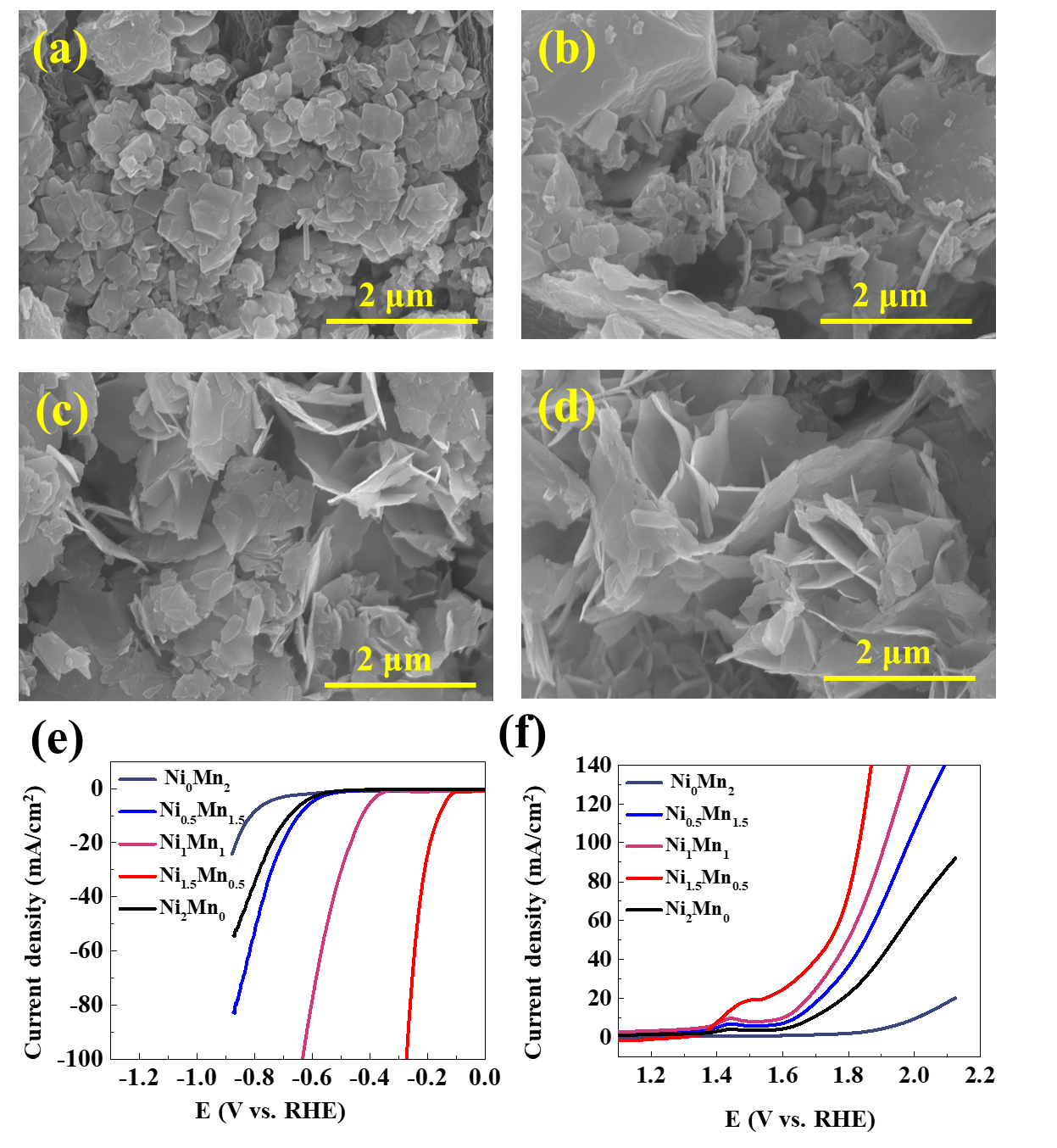
Fig. S6.** (a) - (d) SEM images of Ni_0_Mn_2_, Ni_0.5_Mn_1.5_, Ni_1_Mn_1_ and Ni_2_Mn_0_, (e) - (f) electrochemical performance of Ni-Mn concentration variation for HER, OER.

**
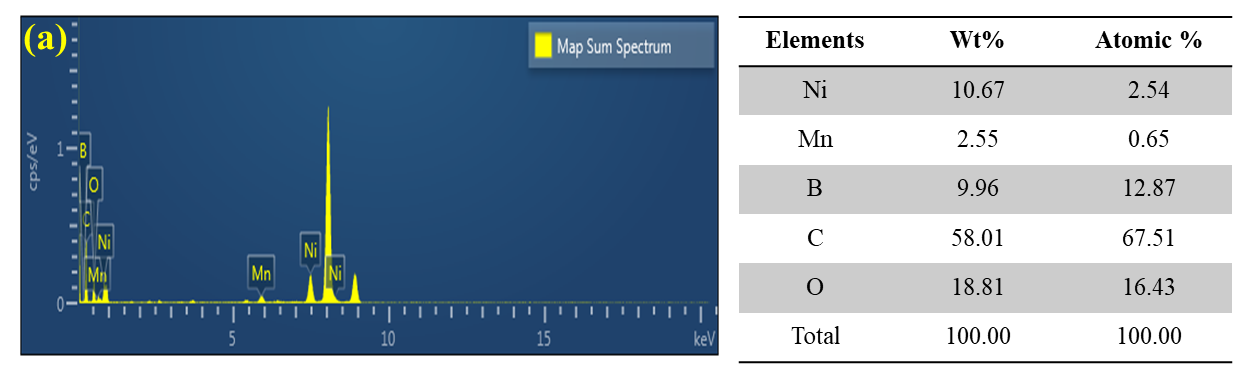
Fig. S7.** (a) EDS spectra and atomic wt % table of rGO/Ni_1.5_Mn_0.5_B.

**
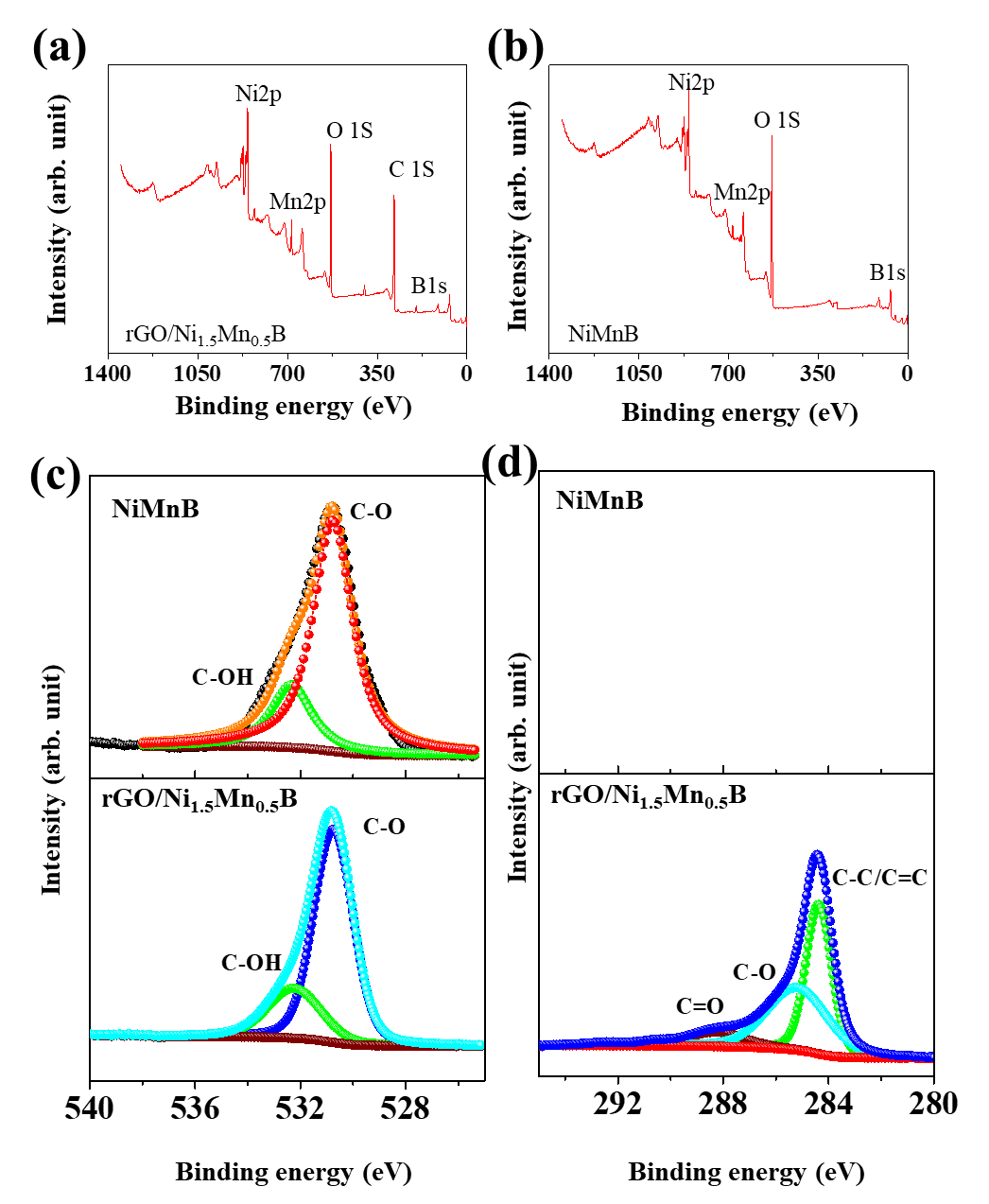
**

**Fig. S8.** XPS analysis (a)-(b) Full scan spectra of rGO/Ni1.5Mn0.5B and NiMnB electrode. (c) - (d) High resolution spectra of NiMnB: O1s and rGO/Ni1.5Mn0.5B: O1s, C 1s.

**
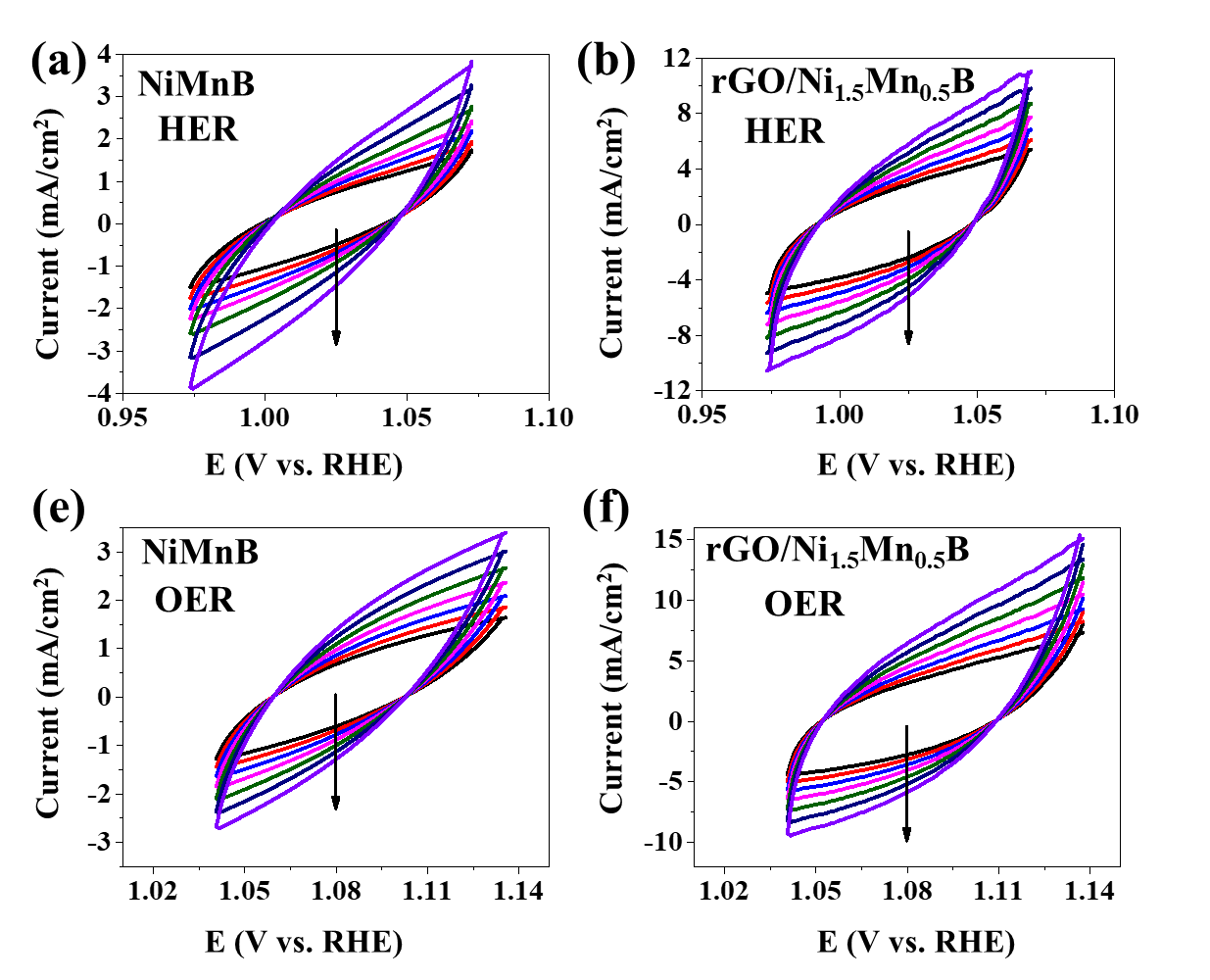
**

**Fig. S9.** Cyclic voltammetry (CV) curve obtained in non- Faradic region for NiMnB (a) HER (e) OER, rGO/Ni_1.5_Mn_0.5_MB (b) HER (f) OER.

**
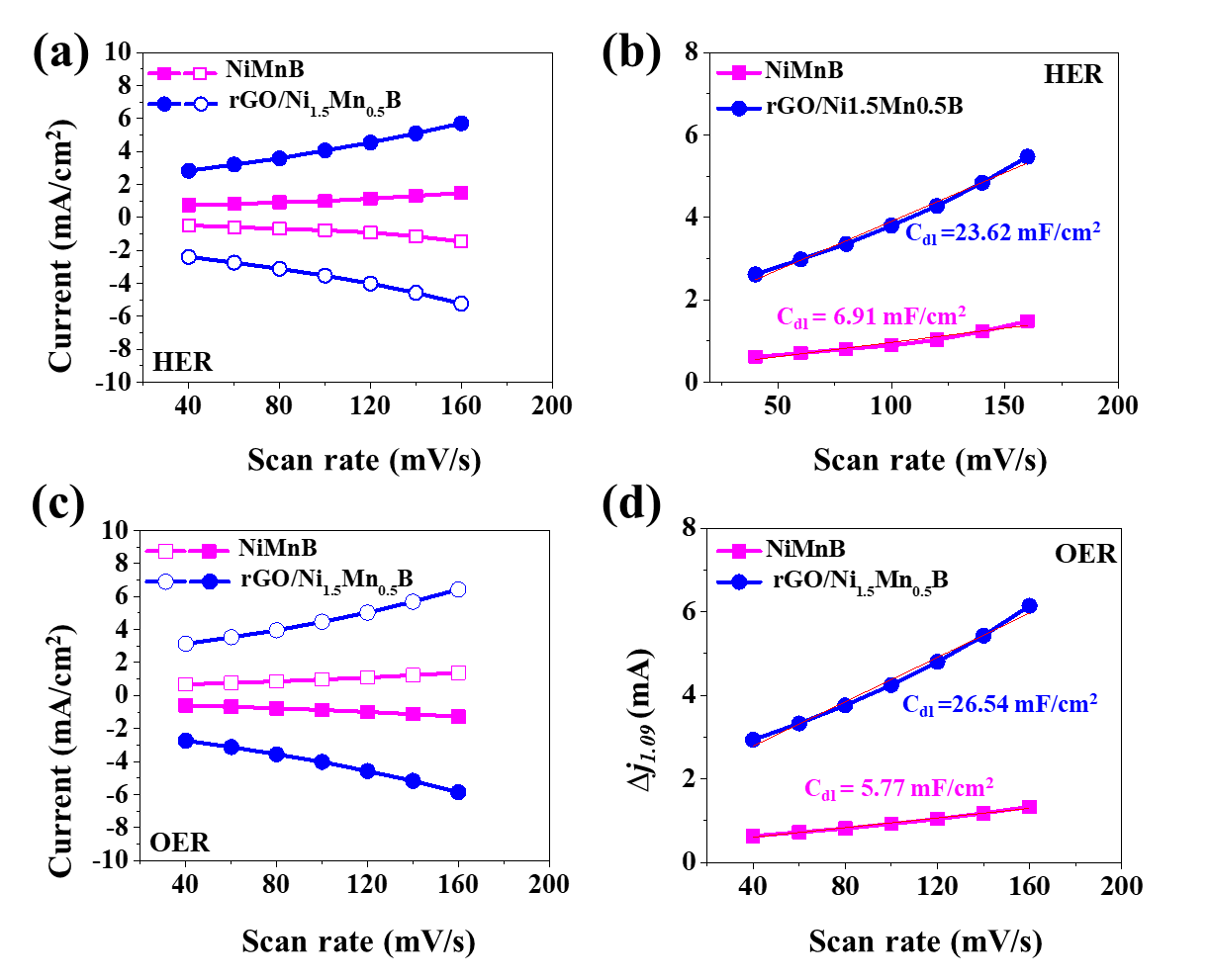
**

**Fig. S10.** (a)-(c)The linear plots for anodic and cathodic current density as a function of scan rate for HER and OER. (b)-(d) Linear plots for HER/OER (versus RHE, *J* = *J*_a_ - *J*_c_)/2) plotted against different scan rates. ^
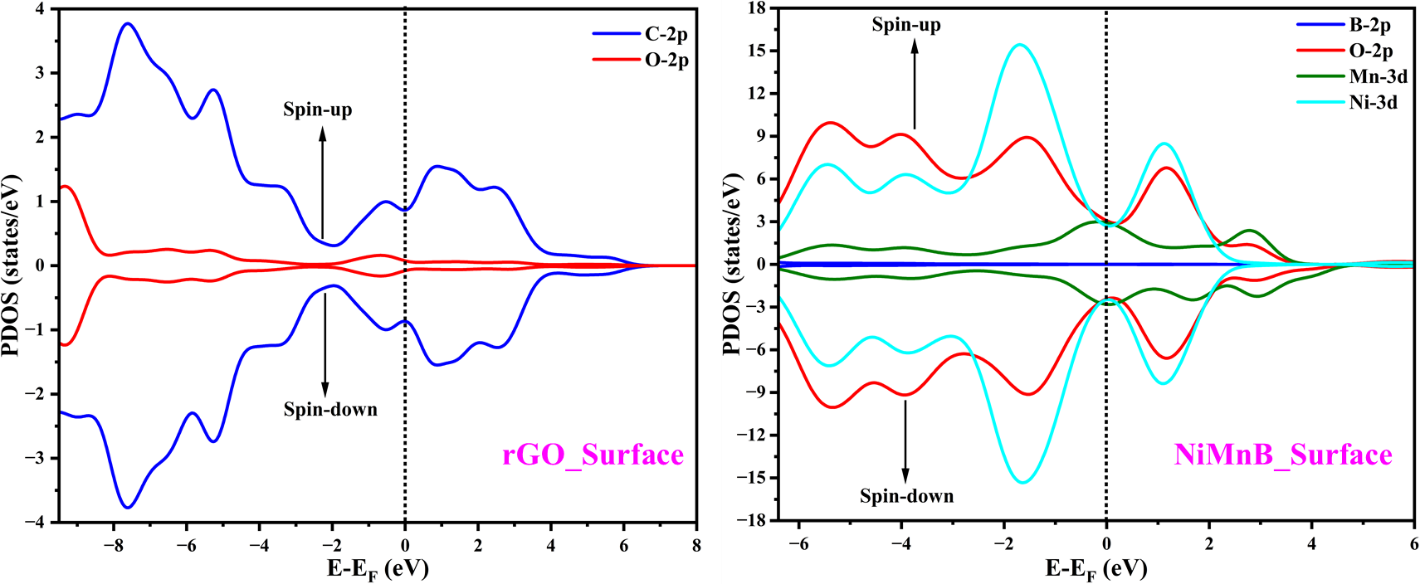
^

**Fig. S11.** Calculated the partial density of states (PDOS) for the rGO and NiMnB surfaces. The Fermi level is set to zero.

^
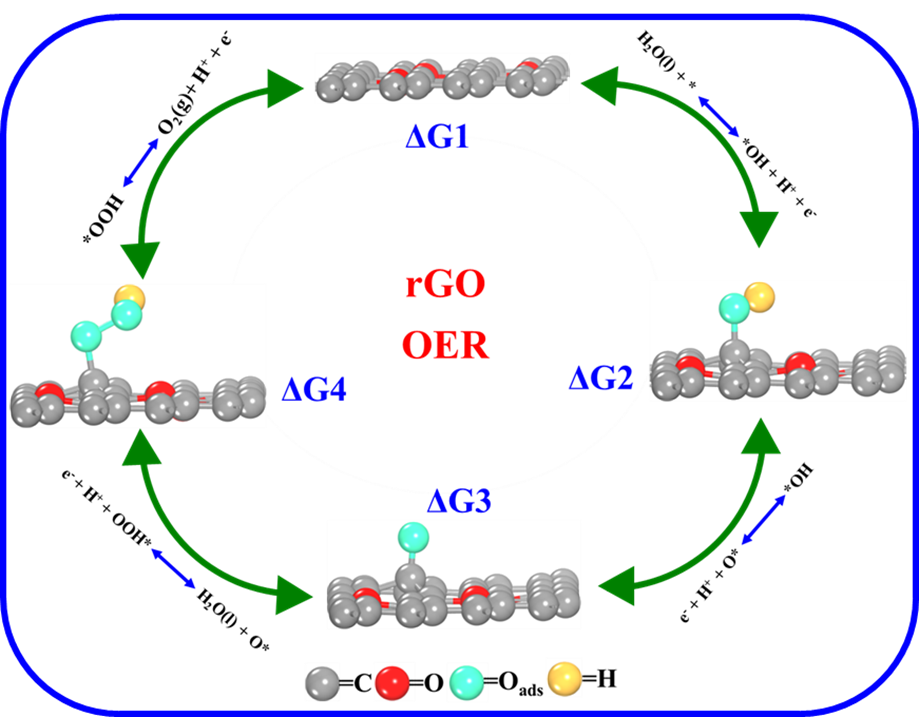
^

**Fig. S12.** Schematic of the 4e^−^ OER pathway of the active site on the rGO surface with the optimized configurations for intermediates.

^
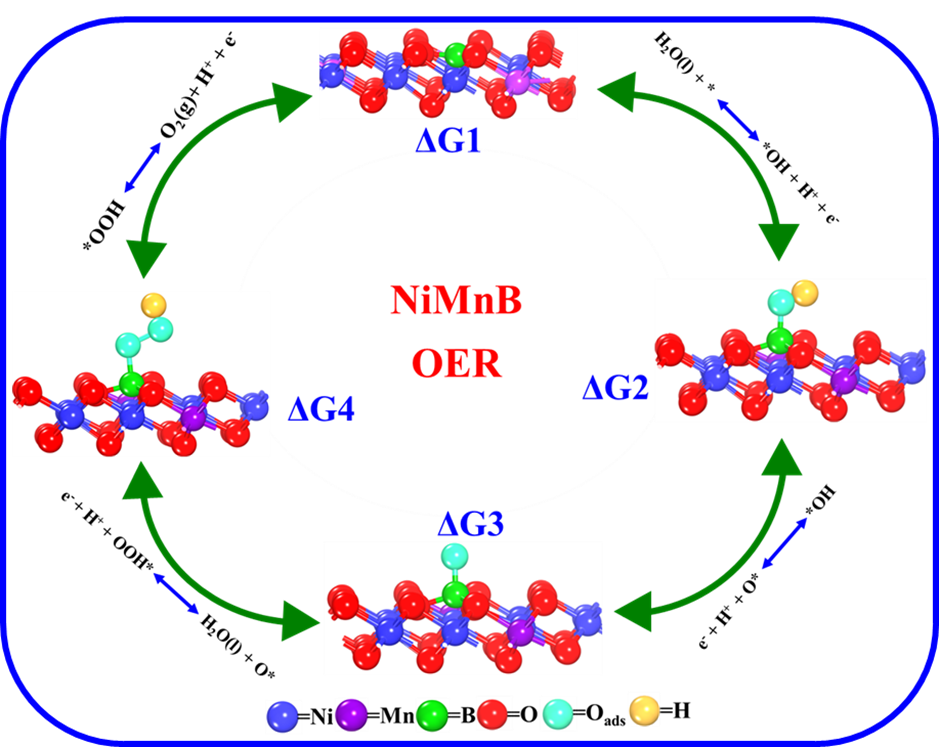
^

**Fig. S13.** Schematic of the 4e^−^ OER pathway of the active site on the NiMnB surface with optimized configurations for intermediates.

**
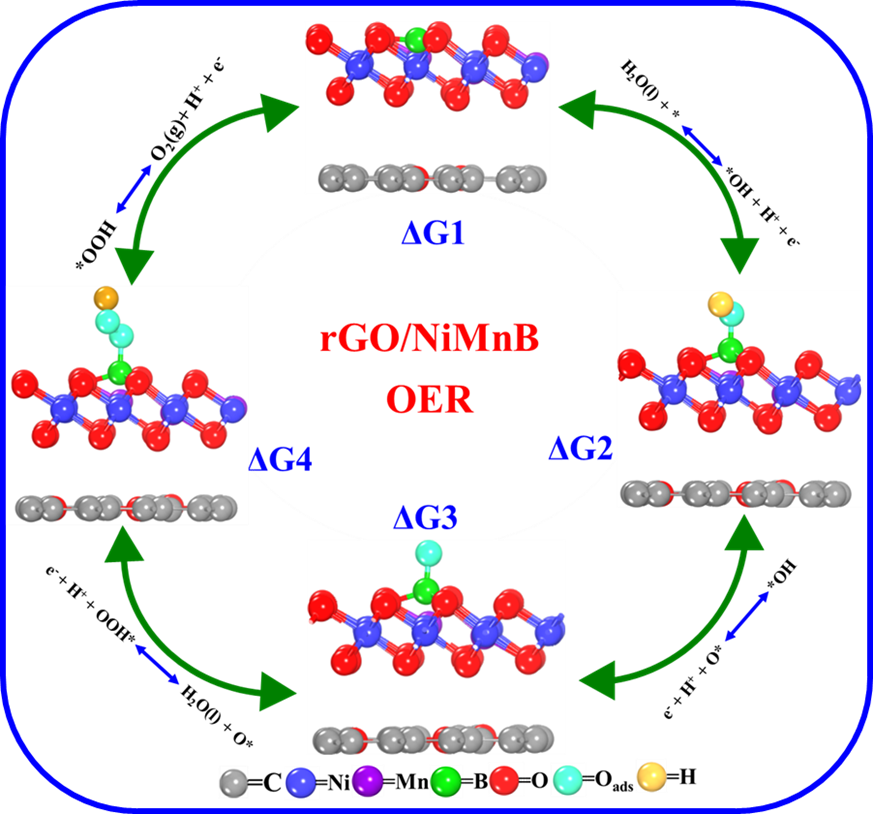
**

**Fig. S14.** Schematic of the 4e^−^ OER pathway of the active site on the rGO/NiMnB hybrid structure with the optimized configurations for intermediates.

**
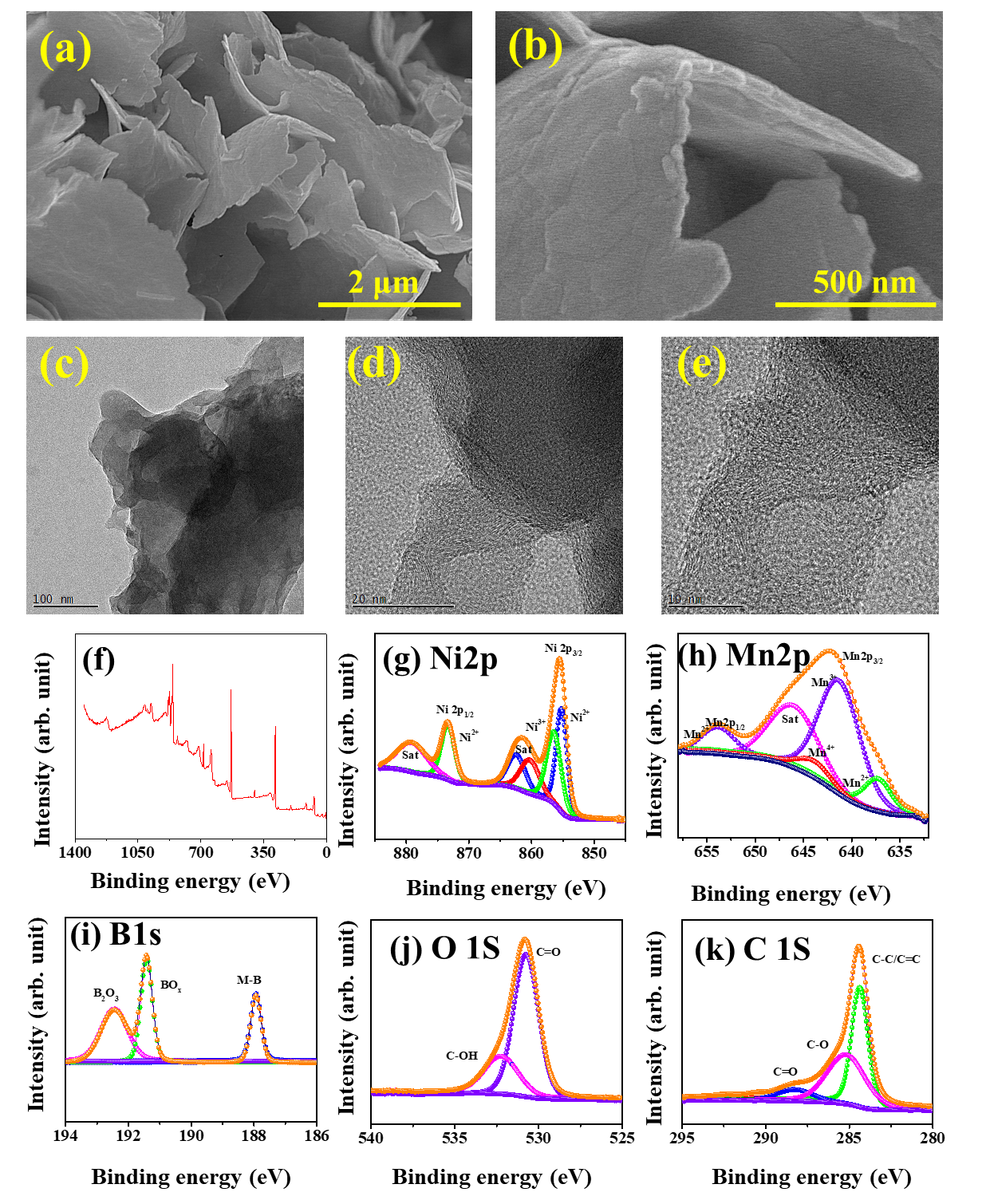
**

**Fig. S15.** After stability test study of rGO/Ni_1.5_M_0.5_B sample. (a) - (b) FE-SEM images. (c)-(d) HRTEM images. (f) Full scan XPS spectra. (g) - (k) High-resolution XPS spectra of Ni 2p, Mn 2p, B 1s, O 1s, C 1s respectively.

**Table S1.** Summary table for HER performance in alkaline electrolyte

| Electrocatalyst | Overpotential  Value (mV) @ 10 mA/cm^2^ | References |
| --- | --- | --- |
| rGO/Ni_1.5_Mn_0.5_B | 159 | This work |
| NiFe@MoS_2_ | 67 | [6] |
| Co–WSe_2_@PANI | 308 | [7] |
| Co_3_O_4_/Ppy/MWCNT | 490 | [8] |
| GC/Ag@CNT | 570 | [9] |
| Ni-N-C | 218 | [10] |
| W-Ni_12_P_5_ | 172 | [11] |
| (Ni, Mn)-CNZ | 288 | [12] |
| HEMP NiCoFeMnCrP NPs | 220 | [13] |
| CGD | 287 | [14] |
| Ni_3_C | 249 | [15] |

**Table S2.** Summary table for OER performance in alkaline electrolyte

| Electrocatalyst | Overpotential  Value (mV) @ 10 mA/cm^2^ | References |
| --- | --- | --- |
| rGO/Ni_1.5_Mn_0.5_B | 170 | This work |
| CoFe@CNT | 260 | [16] |
| Co_3_O_4_/Ppy/MWCNT | 300 | [8] |
| GC/Ag@CNT | 620 | [9] |
| Ni_3_@Fe_7_-N | 361 | [17] |
| Co–WSe_2_@PANI | 360 | [7] |
| NiFe@MoS_2_ | 201 | [6] |
| Co_2_WPB1 | 262 | [18] |
| Ni-N-C | 330 | [10] |
| W-Ni_12_P_5_ | 322 | [11] |
| (Ni, Mn)-CNZ | 380 | [12] |

**Table S3.** Summary table for overall water splitting performance in alkaline electrolyte

| Electrocatalyst | Cell voltage (V)  @ 10 mA/cm^2^ | References |
| --- | --- | --- |
| rGO/Ni_1.5_Mn_0.5_B | 1.49 | This work |
| GO/NiO@CNTs/GO | 1.50 | [19] |
| 1T−Mn−VS_2_@Co_2_P@CC | 1.50 | [20] |
| RuNi@rGO | 1.52 | [21] |
| RuO_2_ – Co_3_O_4_ | 1.54 | [22] |
| FeNi(OH)*_x_*@NF | 1.56 | [23] |
| RuCo−Ti_3_C_2_T*_x_* | 1.56 | [24] |
| HfCoS/rGO | 1.60 | [25] |
| Cu_25_Co_75_P | 1.72 | [26] |
| AL-CNT-CuO-SSE | 1.85 | [27] |
| Co–WSe_2_@PANI | 1.87 | [7] |

**Supporting References**

[1] G. Kresse and J. Furthmüller, Efficiency of ab-initio total energy calculations for metals and semiconductors using a plane-wave basis set, Comput. Mater. Sci. **6**, 15 (1996). <https://doi.org/10.1016/0927-0256(96)00008-0>

[2] P. E. Blöchl, O. Jepsen, and O. K. Andersen, Improved tetrahedron method for Brillouin-zone integrations, Phys. Rev. B **49**, 16223 (1994). https://doi.org/10.1103/PhysRevB.49.16223

[3] J. P. Perdew, J. A. Chevary, S. H. Vosko, K. A. Jackson, M. R. Pederson, D. J. Singh, and C. Fiolhais, Erratum: Atoms, molecules, solids, and surfaces: Applications of the generalized gradient approximation for exchange and correlation, Phys. Rev. B **48**, 4978 (1993). https://doi.org/10.1103/PhysRevB.46.6671

[4] S. Grimme, J. Antony, S. Ehrlich, and H. Krieg, A consistent and accurate ab initio parametrization of density functional dispersion correction (DFT-D) for the 94 elements H-Pu, J. Chem. Phys. **132**, (2010). <https://doi.org/10.1063/1.3382344>

[5] R. Andaveh, A. Sabour Rouhaghdam, J. Ai, M. Maleki, K. Wang, A. Seif, G. Barati Darband, and J. Li, Boosting the electrocatalytic activity of NiSe by introducing MnCo as an efficient heterostructured electrocatalyst for large-current-density alkaline seawater splitting, Appl. Catal. B Environ. **325**, 122355 (2023). <https://doi.org/10.1016/j.apcatb.2022.122355>

[6] Z. Jiang, W. Zhou, C. Hu, X. Luo, W. Zeng, X. Gong, Y. Yang, T. Yu, W. Lei, and C. Yuan, Interlayer‐confined NiFe dual atoms within MoS2 electrocatalyst for ultra‐efficient acidic overall water splitting, Adv. Mater. **35**, 2300505 (2023). <https://doi.org/10.1002/adma.202300505>

[7] S. Cogal, G. C. Cogal, M. Mičušík, M. Kotlár, and M. Omastová, Cobalt-doped WSe_2_@ conducting polymer nanostructures as bifunctional electrocatalysts for overall water splitting, Int. J. Hydrogen Energy **49**, 689 (2024). <https://doi.org/10.1016/j.ijhydene.2023.09.002>

[8] S. S. Jayaseelan, N. Bhuvanendran, Q. Xu, and H. Su, Co_3_O_4_ nanoparticles decorated Polypyrrole/carbon nanocomposite as efficient bi-functional electrocatalyst for electrochemical water splitting, Int. J. Hydrogen Energy **45**, 4587 (2020). <https://doi.org/10.1016/j.ijhydene.2019.12.085>

[9] M. A. Hefnawy, A. Nafady, S. K. Mohamed, and S. S. Medany, Facile green synthesis of Ag/carbon nanotubes composite for efficient water splitting applications, Synth. Met. **294**, 117310 (2023). <https://doi.org/10.1016/j.synthmet.2023.117310>

[10] S. Wu et al., Confined synthesis of highly dispersed Ni anchored on mesoporous carbon as efficient catalyst for water splitting, Mol. Catal. **548**, 113473 (2023). <https://doi.org/10.1016/j.mcat.2023.113473>

[11] S. Ghosh, S. R. Kadam, S. Kolatkar, A. Neyman, C. Singh, A. N. Enyashin, R. Bar-Ziv, and M. Bar-Sadan, W Doping in Ni_12_P_5_ as a Platform to Enhance Overall Electrochemical Water Splitting, ACS Appl. Mater. Interfaces **14**, 581 (2021). <https://doi.org/10.1021/acsami.1c16755>

[12] A. BaQais, M. Shariq, M. A. Qamar, D. Alhasmialameer, A. F. Alharbi, H. A. Althikrallah, M. R. Alrahili, and K. S. Alrashdi, Synthesis and characterization of (Ni, Mn)-ZnO/g-C_3_N_4_ nanocomposite for efficient electrochemical water splitting: The role of electrocatalyst for OER, Diam. Relat. Mater. **147**, 111343 (2024). <https://doi.org/10.1016/j.diamond.2024.111343>

[13] D. Lai, Q. Kang, F. Gao, and Q. Lu, High-entropy effect of a metal phosphide on enhanced overall water splitting performance, J. Mater. Chem. A **9**, 17913 (2021). **DOI** https://doi.org/10.1039/D1TA04755H

[14] B. S. Reghunath, S. Rajasekaran, S. Devi K R, D. Pinheiro, and J. R. Jaleel UC, N-doped graphene quantum dots incorporated cobalt ferrite/graphitic carbon nitride ternary composite for electrochemical overall water splitting, Int. J. Hydrogen Energy **48**, 2906 (2023). <https://doi.org/10.1016/j.ijhydene.2022.10.169>

[15] Y. Huang, H. Zhou, X. Luo, H. Zhan, W. Xu, D. Ye, C. Wu, C. Hu, W. Lei, and C. Yuan, Strain engineering induced surfacial catalytic amorphous Ni_3_C with room temperature ferromagnetism for magnetic heating enhancement of overall water-splitting, Chem. Eng. J. **486**, 150174 (2024). <https://doi.org/10.1016/j.cej.2024.150174>

[16] A. Fatima, H. Aldosari, M. S. Al-Buriahi, M. Al Huwayz, Z. A. Alrowaili, M. S. Alqahtani, M. Ajmal, A. Nazir, M. Iqbal, and R. Tur Rasool, Cobalt Ferrite Surface-Modified Carbon Nanotube Fibers as an Efficient and Flexible Electrode for Overall Electrochemical Water Splitting Reactions, ACS Omega **8**, 37927 (2023). <https://doi.org/10.1021/acsomega.3c03314>

[17] S. Mathi and J. Jayabharathi, Enhanced stability and ultrahigh activity of amorphous ripple nanostructured Ni-doped Fe oxyhydroxide electrode toward synergetic electrocatalytic water splitting, RSC Adv. **10**, 26364 (2020). **DOI** https://doi.org/10.1039/D0RA04828C

[18] A. Bhide, S. Gupta, R. Bhabal, K. H. Mali, B. R. Bhagat, A. Dashora, M. Patel, R. Fernandes, and N. Patel, Unveiling the synergistic effect of amorphous CoW-phospho-borides for overall alkaline water electrolysis, Int. J. Hydrogen Energy **63**, 645 (2024). <https://doi.org/10.1016/j.ijhydene.2024.03.090>

[19] S. Alotibi, A. Khalid, E. G. Hanna, Z. M. Aldhafeeri, M. Hasan, T. Al Haq, and A. Ali, Fabrication of nickel oxide decorated CNTs/GO nanohybrid: A multifunctional electrocatalyst for overall electrochemical water splitting, FlatChem **48**, 100732 (2024). <https://doi.org/10.1016/j.flatc.2024.100732>

[20] P. P. Dhakal, U. N. Pan, M. R. Kandel, R. B. Ghising, T. H. Nguyen, V. A. Dinh, N. H. Kim, and J. H. Lee, Cobalt phosphide integrated manganese-doped metallic 1T-vanadium disulfide: Unveiling a 2D-2D tangled 3D heterostructure for robust water splitting, Chem. Eng. J. **473**, 145321 (2023). <https://doi.org/10.1016/j.cej.2023.145321>

[21] M. Yang, J. Wang, P. Dai, X. Tang, G. Li, and L. Yang, RuNi single-atom alloy anchored on rGO as an outstanding bifunctional catalyst for efficient electrochemical water splitting, New J. Chem. **48**, 3942 (2024). DOI https://doi.org/10.1039/D3NJ05436E

[22] F. Ren, J. Xu, and L. Feng, An effective bimetallic oxide catalyst of RuO_2_-Co_3_O_4_ for alkaline overall water splitting, Nano Res. **17**, 3785 (2024). https://doi.org/10.1007/s12274-023-6316-4

[23] B. Li, J. Zhao, Y. Wu, G. Zhang, H. Wu, F. Lyu, J. He, J. Fan, J. Lu, and Y. Y. Li, Identifying Fe as OER active sites and ultralow‐cost bifunctional electrocatalysts for overall water splitting, Small **19**, 2301715 (2023). https://doi.org/10.1002/smll.202301715

[24] X. Yu, L. Lin, C. Pei, S. Ji, Y. Sun, Y. Wang, J. Kyu Kim, H. Seok Park, and H. Pang, Immobilizing Bimetallic RuCo Nanoalloys on Few‐Layered MXene as a Robust Bifunctional Electrocatalyst for Overall Water Splitting, Chem. Eur. J. **30**, e202303524 (2024). https://doi.org/10.1002/chem.202303524

[25] M. Itagi, D. Chauhan, and Y.-H. Ahn, HfCoS/rGO bifunctional electrocatalysts for efficient water splitting in alkaline media, Energy & Fuels **37**, 11298 (2023). <https://doi.org/10.1021/acs.energyfuels.3c01336>

[26] D. Bandyopadhyay, S. Ghosh, L. Houben, R. Bar-Ziv, and M. Bar-Sadan, Full Water Splitting Electrolyzed by Cu–Co Bimetallic Phosphides, ACS Appl. Energy Mater. **6**, 10987 (2023). <https://doi.org/10.1021/acsaem.3c01761>

[27] M. F. Sanad, V. S. N. Chava, T. Zheng, S. Pilla, B. Joddar, and S. T. Sreenivasan, Unraveling the Cooperative Activity of Hydrophilicity, Conductivity, and Interfacial Active Sites in Alginate‐CNT‐Cuo Self‐Standing Electrodes with Benchmark‐Close Activity for Alkaline Water Splitting, Adv. Sustain. Syst. **7**, 2300283 (2023). **https://doi.org/10.1002/adsu.202300283**
